# Fine‐Tuning Photochemical Immunogenic Cell Death by a Panel of Verteporfin‐Lipid Nanoparticles: A Data‐Driven Approach

**DOI:** 10.1002/smsc.202500290

**Published:** 2025-10-05

**Authors:** Nimit Shah, Maxwell Bortei Quaye, Siddharth Reddy Soma, Meghana Sree Vadlamudi, Doha Mahmoud, Ashritha Malkoochi, Taksheel Rao Aileni, Chanda Bhandari, Kunal Karambelkar, Tayyaba Hasan, Mladen Korbelik, Baowei Fei, Xinxin Song, Girgis Obaid

**Affiliations:** ^1^ Department of Bioengineering The University of Texas at Dallas Richardson TX 75080 USA; ^2^ Wellman Center for Photomedicine Massachusetts General Hospital Harvard Medical School Boston MA 02114 USA; ^3^ Division of Health Sciences and Technology Harvard University Massachusetts Institute of Technology Cambridge MA 02139 USA; ^4^ Department of Integrative Oncology BC Cancer Vancouver British Columbia V5Z 1L3 Canada; ^5^ Department of Radiology UT Southwestern Medical Center Dallas TX 75390 USA; ^6^ Department of Surgery UT Southwestern Medical Center Dallas TX 75390 USA

**Keywords:** benzoporphyrin derivative, immunogenic cell death, liposomes, photodynamic therapy, principal component analysis, reactive oxygen species, solid lipid nanoparticles

## Abstract

Immunogenic cell death (ICD) is an immunostimulatory process that can be induced by light‐activated photosensitizers, but its mechanisms remain unclear, especially with lipid nanoparticle (LNP) formulations. In this study, a multivariate, data‐driven analysis was conducted using a panel of five verteporfin(V)‐LNPs to identify the attributes that lead to the greatest photochemically‐induced exposure of ICD markers in pancreatic cancer cells. These attributes include varying production of Type I (radicals) or Type II (singlet oxygen) reactive oxygen species (ROS) upon 690 nm activation, localization in different organelles, variable cellular uptake efficiencies, and different phototoxicity levels. Using principal component analysis, we identified that, unexpectedly, Type I ROS is most strongly associated with ICD marker exposure, which leads to dendritic cell activation ex vivo, while Type II ROS shows the weakest association. Furthermore, V‐LNP localization in the endoplasmic reticulum and mitochondria is most strongly associated with exposure of ICD markers, while lysosomal localization shows the weakest association. ICD marker exposure is proportional to the degree of phototoxicity and cellular uptake efficiency for all V‐LNPs. These findings provide critical insights into the multiparametric mechanism underlying photochemical ICD induced by V‐LNPs and can inform the rational design of photochemical LNP constructs for augmenting anticancer immune responses.

## Graphical abstract

The relationship between the attributes of verteporfin‐lipid nanoparticles and the exposure of immunogenic cell death markers (HSP‐70, HMGB1, and calreticulin) following 690 nm light activation in murine pancreatic cancer cells.

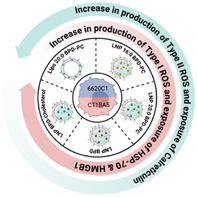

## 1 Introduction

Immunogenic cell death (ICD) is a critical immunostimulatory process that leads to the activation of innate immune cells and is the first step of the antitumor immunity cycle.^[^ ^1^, ^2^, ^3^, ^4^ ^]^ Photochemical stimulation during photodynamic therapy (PDT) has been suggested to be one of the most effective methods for inducing ICD in cancer cells, and can be superior to chemotherapeutics such as mitoxantrone or doxorubicin.^[^ ^1^, ^5^, ^6^ ^]^ Photochemical stimulation involves the activation of photosensitizers using light to generate reactive oxygen species (ROS).^[^ ^7^, ^8^ ^]^ Depending on the dose used, photochemical stimulation can be cytotoxic or can “prime” cancer cells and/or the tumor microenvironment for subsequent treatments.^[^ ^4^, ^8^, ^9^, ^10^ ^]^ The degree of photochemically‐induced cell death is influenced by multiple factors including the amount of ROS production, subcellular localization of the photosensitizer and its local concentration, and the light irradiation parameters.^[^ ^11^ ^]^ Traditionally, necrosis,^[^ ^10^ ^]^ autophagy,^[^ ^12^ ^]^ and apoptosis,^[^ ^13^ ^]^ have been recognized as the primary cell death pathways following photochemical stimulation. However, recent studies have identified several other pathways involved, such as ferroptosis,^[^ ^14^, ^15^ ^]^ immunogenic necroptosis,^[^ ^16^ ^]^ pyroptosis,^[^ ^17^ ^]^ and paraptosis,^[^ ^18^ ^]^ and these pathways have also been implicated in photochemical ICD.^[^ ^1^, ^4^, ^11^, ^19^, ^20^ ^]^ Following photochemical stimulation, stressed or dying cells can release or translocate damage‐associated molecular patterns (DAMPs) to the cell surface, such as calreticulin,^[^ ^21^, ^22^, ^23^ ^]^ heat shock protein (HSP)‐70,^[^ ^21^, ^22^, ^24^ ^]^ and high mobility group box 1 (HMGB1),^[^ ^24^ ^]^ among others.^[^ ^25^, ^26^, ^27^, ^28^, ^29^, ^30^ ^]^ These DAMPs facilitate the recruitment of antigen‐presenting cells like macrophages and dendritic cells (DCs) which initiate a cascade of immune cell responses that can ultimately lead to antitumor T‐cell adaptive responses and immune memory.^[^ ^22^, ^23^, ^31^, ^32^ ^]^

Although many nanoparticles, including lipid nanoparticles (LNP), have been used to induce photochemical ICD of cancer cells,^[^ ^24^, ^28^, ^29^, ^30^, ^33^, ^34^ ^]^ the mechanisms by which they do so are not fully understood. Without a deep mechanistic understanding of how nanoparticle‐based regimens are responsible for the efficient induction of photochemical ICD, the rational design of such nanoparticles and treatment protocols cannot be accurately tailored for cancer immunotherapy applications. For example, the impact of the type of photochemically generated ROS on the efficiency of ICD has not been studied. While some Type I dominant photosensitizer‐containing nanoparticles have been reported to induce ICD,^[^ ^33^, ^34^ ^]^ other Type II dominant photosensitizer‐containing nanoparticles have also been reported to induce ICD.^[^ ^35^, ^36^, ^37^ ^]^ Likewise, the role of subcellular localization in photochemical ICD is not fully understood. Various reports in the literature have identified direct endoplasmic reticulum (ER) photodamage as being critical for ICD,^[^ ^38^, ^39^ ^]^ while others demonstrate efficient ICD induction using photosensitizers that exclusively localize in the lysosomes.^[^ ^1^, ^24^, ^33^ ^]^ Lastly, the relationship between the degree of cancer cell utpake and phototoxicity and the efficiency of photochemical ICD induction also remains poorly understood. Some studies suggest that low, noncurative PDT protocols enhance antitumor immune responses in vivo,^[^ ^30^ ^]^ while others report efficient T‐cell responses and abscopal effects using high, curative PDT doses.^[^ ^40^ ^]^

To the best of our knowledge, this is the first systematic and comprehensive study to investigate the interrelationship between the type of photochemical reactions, the uptake efficiency, the subcellular localization, and the degree of phototoxicity on the efficiency of photochemical ICD induction with nanoparticle‐based regimens using LNPs as a model system.

**Scheme 1:**
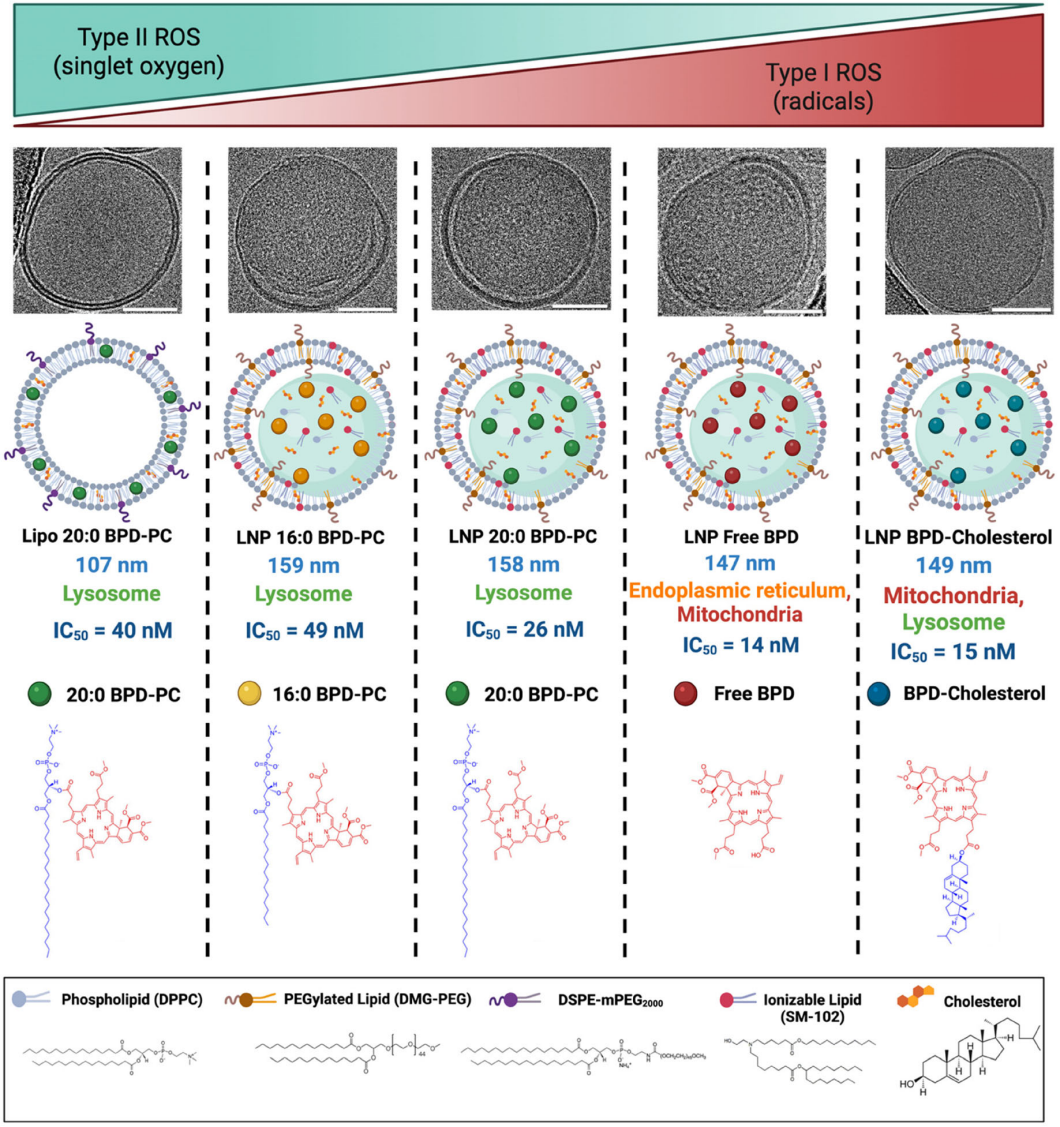
Cryo‐TEM images and graphical representations of the V‐LNPs used in this study, including liposomes as a control. Verteporfin (BPD) is formulated in its native or conjugated form when tethered to the lipids 16:0 PC, 20:0 PC, or cholesterol. The V‐LNPs vary in their production of Type I (radicals) and Type II (singlet oxygen) ROS, their cellular uptake efficiencies, their subcellular localization, and their degree of phototoxicity. (Scale bars in electron microscopy images are 50 nm).

**Scheme 2:**
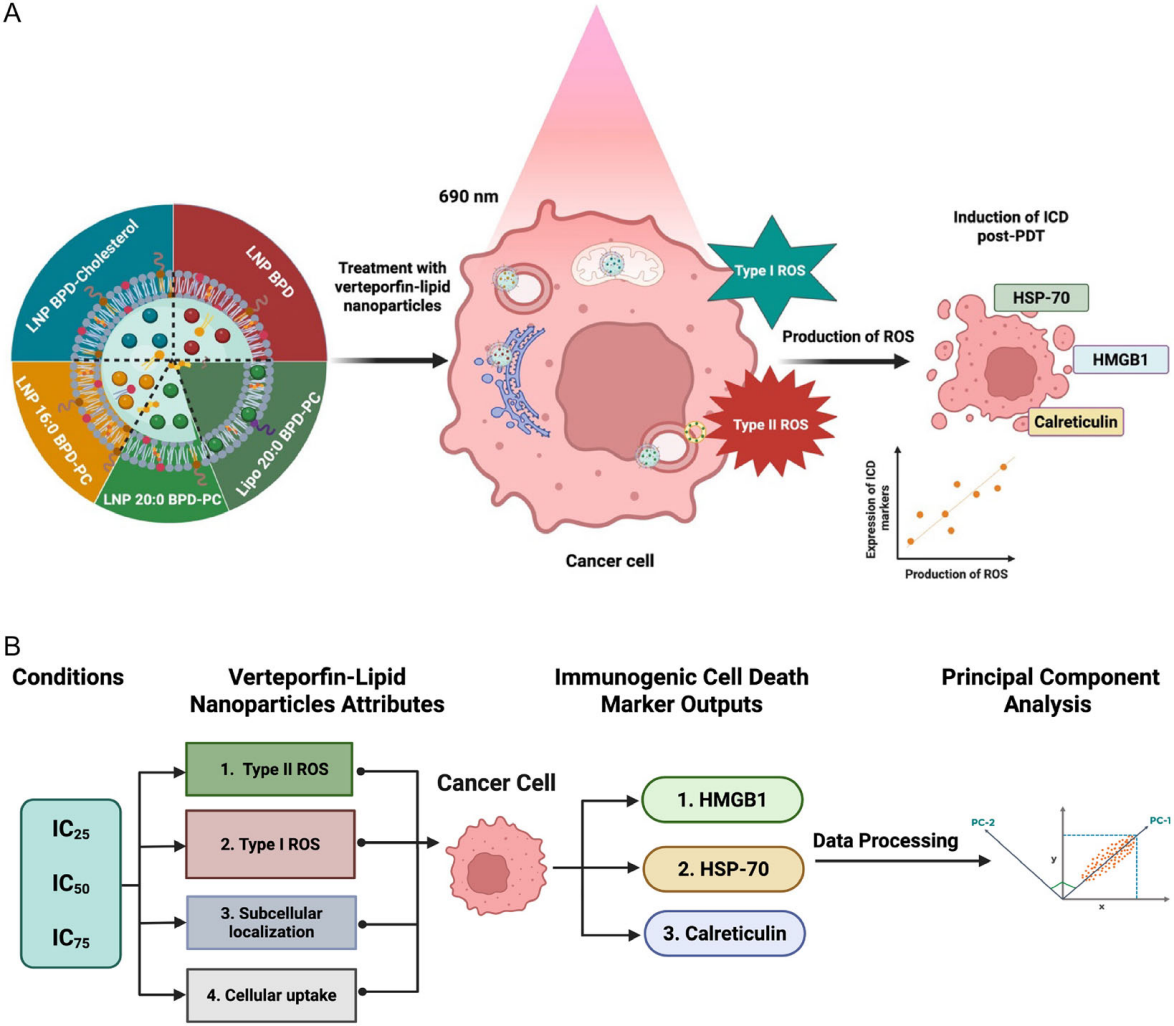
A) Graphical representation of photochemical ICD using V‐LNPs in pancreatic cancer cells that localize in various organelles with various uptake efficiencies, produce varying levels of Type I and Type II ROS, and exhibit varying degrees of phototoxicity. B) A flowchart describing the workflow of data processing using PCA for photochemically‐induced ICD using V‐LNPs, which evaluates the impact of the degree of V‐LNP phototoxicity, ROS production, cellular uptake efficiency, and subcellular localization on the exposure of ICD markers.

Visudyne, a liposomal formulation of the hydrophobic photosensitizer verteporfin, is approved for PDT of wet age‐related macular degeneration and is being evaluated in clinical trials for pancreatic cancer, among others (e.g., NCT03033225, NCT02872064, and NCT06381154).^[^ ^41^, ^42^ ^]^ Although Visudyne effectively solubilizes verteporfin, it rapidly leaks out of the formulation in the presence of serum.^[^ ^43^, ^44^, ^45^, ^46^ ^]^ We have shown that conjugating verteporfin to various lipids reduces this leakage in serum.^[^ ^29^, ^30^, ^43^, ^44^, ^47^, ^48^ ^]^ In this study, we formulated verteporfin (chemical name: benzoporphyrin derivative (BPD)) and verteporfin conjugated to lipids (16:0 lyso PC, 20:0 lyso PC, or cholesterol) into solid LNPs and liposomes as a control. This yielded a panel of verteporfin (V)‐LNPs with distinct attributes that we evaluate in the context of photochemical ICD (**Scheme** **1**). These attributes include a broad range of efficiencies of Type I (radical) and Type II (singlet oxygen) photochemical reactions, cellular uptake, different patterns of subcellular localization in mouse pancreatic cancer cells, and varying degrees of phototoxicity. Given the large number of variables analyzed, we leverage principal component analysis (PCA) in this study to reduce the high dimensionality of the datasets in order to identify the key V‐LNP attributes that are most closely associated with the exposure of ICD markers HMGB1, HSP‐70, and calreticulin following activation with 690 nm light (**Scheme** **2**). This data‐driven study reveals previously unidentified mechanistic insights into V‐LNP design features that can be fine‐tuned to augment photochemical ICD in cancer cells and may provide a framework for the rational design of other photosensitizer‐encapsulated nanoparticles. The impact of fine‐tuning photochemical ICD by V‐LNPs on the innate and adaptive immune contexture in tumors, as well as DC activation, T‐cell education, and cooperation with immune checkpoint inhibitors, is the focus of upcoming studies.

## 2 Results and Discussion

### 2.1 Synthesis and Characterization of V‐LNPs

The synthesis procedures for the verteporfin‐lipid conjugates and the V‐LNPs are described in the supplementary Methods section. The hydrodynamic diameters, polydispersity indices (PDIs), and *ζ*‐potentials of all the V‐LNPs are summarized in **Table** **1**. The hydrodynamic diameters of the V‐LNPs were all within the size range of 100–180 nm, which is common for LNPs.^[^ ^29^, ^49^, ^50^ ^]^ Dynamic light scattering (DLS) analysis confirmed the uniformity of the V‐LNP formulations, as evidenced by their PDI values. In nanoparticle characterization, a PDI value of <0.2 is generally indicative of monodisperse particles. All V‐LNP formulations in this study exhibited PDI values below 0.2, confirming their monodispersity and uniform size distribution. The *ζ*‐potentials of the V‐LNPs were between −1.13 and −4.30 mV, which were anticipated to be close to neutral because the ionizable lipid SM‐102 remains uncharged in neutral buffer.^[^ ^51^, ^52^ ^]^ The physicochemical stability of V‐LNPs during storage in DPBS at 4 °C and during incubation in serum‐containing media at 37 °C was also measured using DLS. All V‐LNPs remained relatively stable under all conditions for up to 7 days (Figure S2, Supporting Information).

**Table 1:** A summary of the physicochemical properties of the V‐LNPs used in this study. (Values are mean ± S.D., *n* = 3).

V‐LNPs	Hydrodynamic diameter [nm ± S.D.]	Polydispersity index [P.D.I. ± S.D.]	*ζ*‐Potential [mV ± S.D.]
Lipo 20:0 BPD‐PCa)	107 ± 1.0	0.13 ± 0.01	−1.56 ± 0.13
LNP 20:0 BPD‐PCa)	158 ± 1.0	0.09 ± 0.02	−4.30 ± 0.18
LNP 16:0 BPD‐PC	159 ± 0.5	0.10 ± 0.01	−1.13 ± 0.17
LNP BPD	147 ± 1.0	0.10 ± 0.01	−1.89 ± 0.18
LNP BPD‐Cholesterol	149 ± 0.9	0.09 ± 0.01	−1.37 ± 0.12

a) Data adapted from our recently published study.^[^ ^29^ ^]^

Using cryo‐transmission electron microscopy (cryo‐TEM) we visualized the morphology of the V‐LNPs. As shown in Scheme 1 and Figure S1, Supporting Information, the micrographs reveal that LNP 20:0 BPD‐PC, LNP 16:0 BPD‐PC, LNP BPD, and LNP BPD‐Cholesterol exhibit a well‐defined structure, consisting of amorphous lipid aggregates coated with partially fused mono‐, bi‐, and multilipid layers. These observations are in accordance with a study in the literature on LNPs containing ionizable lipids (such as SM‐102 that we use here), which showed that PEG‐lipids, DSPC, and cholesterol can partition to an outer lipid layer, while the ionizable lipids accumulate in the LNP interior as an amorphous aggregate.^[^ ^53^ ^]^ In contrast to the solid LNP structures, Lipo 20:0 BPD‐PC exhibits well‐organized bilayers with a hollow aqueous interior, which is consistent with typical liposome structures that we have shown before.^[^ ^48^ ^]^

The optical properties of the V‐LNPs were also studied. Figure S3A, Supporting Information, shows the raw absorbance spectra of all the V‐LNPs at 5 μM verteporfin‐equivalent concentrations in DPBS. The solid LNP formulations all exhibited more light scattering than the Lipo 20:0 BPD‐PC. Larger nanoparticles are known to scatter more light, which could account for the differences we observed.^[^ ^54^, ^55^ ^]^ As shown in Figure S3B–E, Supporting Information, by accounting for the effects of scattering, we back‐calculated the molar extinction coefficients of all the V‐LNPs using the Beer–Lambert Law, as we previously described.^[^ ^24^ ^]^ Verteporfin and its lipid conjugates used in this study have an extinction coefficient of *ε*_687_ nm = 34 895 M^−^^1^ cm^−^^1^ in DMSO as they all dissolve fully in the solvent.^[^ ^44^ ^]^ The molar extinction coefficient of verteporfin and its lipid conjugates formulated in the V‐LNPs is reduced to 21 032–34 460 M^−^^1^ cm^−^^1^ in DPBS, which is likely attributed to varying degrees of J‐aggregates within the formulations that dampen the Q‐bands of the verteporfin variants in all the V‐LNPs.^[^ ^56^, ^57^, ^58^, ^59^, ^60^, ^61^ ^]^ The reductions in the molar extinction coefficients of verteporfin and its lipid conjugates when formulated in V‐LNPs also coincided with reductions in fluorescence emission intensities in DPBS and in serum. As shown in Figure S4A,B, Supporting Information, LNP BPD exhibited the highest fluorescence emission intensity in both DPBS and serum, while Lipo 20:0 BPD‐PC was lowest. This observation aligns with the literature and our previous findings, suggesting that face‐to‐face stacking and static quenching of porphyrins, including verteporfin‐lipid conjugates, when entrapped in liposomal bilayers, leads to lower fluorescence emission intensities, as compared to their disordered arrangement within the solid LNPs.^[^ ^57^, ^59^ ^]^

We also assessed the photostability of the V‐LNPs during 690 nm light irradiation in DPBS and in serum (Figure S5, Supporting Information). ROS produced during 690 nm light irradiation degrades the photosensitizer into photoproducts that cannot be used for photochemical ICD. ≈50% of photoactivity was retained in all V‐LNPs after 20 J cm^−2^ of 690 nm light irradiation in DPBS and in serum, and the differences between the different V‐LNPs did not exceed 10% photobleaching. As such, all V‐LNPs remained up to 50% photoactive using the light activation parameters that were able to induce ICD marker exposure and elicit up to 100% cellular phototoxicity. For context, we have previously shown that Lipo 20:0 BPD‐PC is approximately twice as photostable as the clinical formulation of verteporfin, Visudyne,^[^ ^44^ ^]^ which is capable of effectively inducing patient PDAC tumor tissue necrosis in clinical trials.^[^ ^62^, ^63^ ^]^

### 2.2 Mouse Pancreatic Cancer Cell Lines

In this study, we evaluated photochemical ICD by V‐LNPs in CT1BA5 cells and in 6620c1 cells. CT1BA5 cells are derived from a genetically engineered mouse model of PDAC developed from the KPC mouse model (*Kras* ^LSL−G12D^; *Trp53* ^f l/f l^; *PDX* ^Cre/+^) by the Brekken Lab (UT Southwestern Medical Center). Using whole tumor flow cytometry of subcutaneous CT1BA5 tumors, we demonstrated that these tumors are non‐T cell inflamed with <1% tumor infiltration of CD8^+^ T cells at baseline (Figure S16C, Supporting Information). The 6620c1 tumor model was developed by the Stanger Lab (University of Pennsylvania), which presents a T cell‐inflamed (immune “hot”) phenotype with robust CD8^+^ T‐cell infiltration (≈8% at baseline).^[^ ^64^ ^]^ These two PDAC cancer cell lines are used to set the stage for future in vivo studies to evaluate immune responses using light‐activated V‐LNPs in mouse PDAC models with immunologic heterogeneity.

### 2.3 Quantifying Type I and Type II ROS Production by the V‐LNPs (Input Metric for PCA)

**Figure 1:**
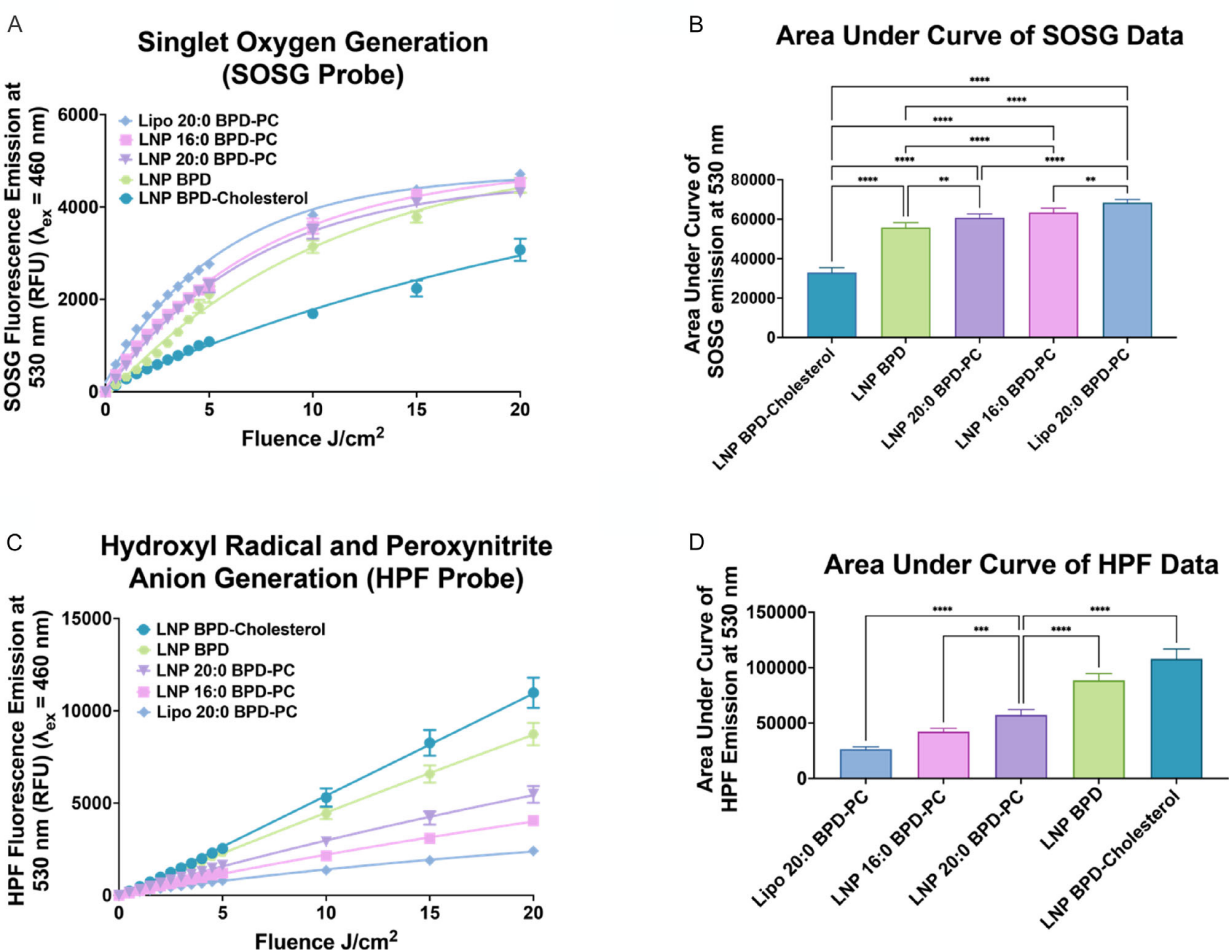
Generation of ROS by V‐LNPs measured using fluorescent probes. A,B) Singlet oxygen production measured using the fluorescent probe SOSG, *λ*_Exc_ = 460 nm and *λ*_Emi_ = 530 nm. C,D) Hydroxyl radical and/or peroxynitrite anion generation measured using the fluorescent probe HPF, *λ*_Exc_ = 460 nm and *λ*_Emi_ = 530 nm. (All data are presented as mean ± S.D., statistical significance was calculated using a one‐way ANOVA test using GraphPad Prism v10.4.1, *: *P* < 0.1, **: *P* < 0.01, ***: *P* < 0.001, ****: *P* < 0.0001).

Photosensitizers undergo varying levels of Type I and Type II photochemical reactions upon excitation by light to elicit therapeutic effects during PDT.^[^ ^65^, ^66^ ^]^ Type I reactions can produce radical‐based ROS, such as hydroxyl radicals and superoxide anion, by electron transfer from excited triplet state photosensitizers. Type II reactions produce a nonradical based ROS, singlet oxygen, through triplet–triplet energy transfer from excited triplet state photosensitizers to ground state triplet oxygen. Most clinical PDT agents are Type II dominant photosensitizers, and a high singlet oxygen quantum yield is typically considered advantageous.^[^ ^8^, ^67^, ^68^ ^]^ On the other hand, Type I dominant photosensitizers are frequently developed with the primary goal of antitumor activity in hypoxia, as they can be less dependent on oxygen for their therapeutic effects during PDT when these photosensitizers react directly with cellular substrates.^[^ ^33^, ^69^, ^70^, ^71^ ^]^ However, very little is known about the role of the type of photochemical reactions during PDT on the efficiency of ICD induction in cancer cells. Recent studies reported the development of Type I ROS‐generating nanoparticles that effectively promoted the exposure of DAMPs, including HMGB1, HSP70, and extracellular ATP, in 4T1 murine breast cancer cells.^[^ ^33^, ^34^ ^]^ On the other hand, another study reported that pyroptosis induced by Type II ROS‐generating liposomes leads to the increased exposure of calreticulin, HMGB1, and extracellular ATP release in 4T1 murine breast cancer cells.^[^ ^37^ ^]^ In this study, we quantified the amount of Type I and Type II ROS generated by the V‐LNPs and used these values as input metrics for the subsequent PCA analysis to identify the combinations of V‐LNP attributes that are most closely associated with exposure of ICD markers. As shown in **Figure** **1**, singlet oxygen generation was measured using the fluorescent Singlet Oxygen Sensor Green (SOSG) probe, and hydroxyl radical and/or peroxynitrite anion generation was measured using the fluorescent hydroxyphenyl fluorescein (HPF) probe. Lipo 20:0 BPD‐PC generated the greatest amount of singlet oxygen, which was two‐fold higher than LNP BPD‐Cholesterol. Conversely, LNP BPD‐Cholesterol generated the greatest amount of hydroxyl radicals and/or peroxynitrite anion, which was four‐fold greater than for Lipo 20:0 BPD‐PC, highlighting an inverse trend between Type II and Type I ROS generation for the V‐LNPs. While photosensitizing systems with the singlet oxygen quantum yield (Type II ROS) is typically the goal in the field of PDT, here we note that lower singlet oxygen production by V‐LNPs corresponded to a greater production of Type I ROS. As such, there is no notable compromise in the photochemical activity of V‐LNPs, but rather a switching of photochemical reaction pathways, as demonstrated in the correlation heatmap (Figure 5) where Type I and Type II ROS are inversely correlated with a significant Pearson's r correlation coefficient of −0.886. We hypothesize that this shift in photochemistry from Type II to Type I ROS is attributed to the presence of the electron‐rich ionizable lipid SM‐102 in the V‐LNPs.^[^ ^29^, ^72^, ^73^ ^]^ However, elucidating the exact mechanisms behind the differences in ROS production between the V‐LNPs would require more in‐depth photophysical analyses, which are part of ongoing and future studies.

As shown in the confocal microscopy images in Figure S6, Supporting Information, intracellular ROS production in CT1BA5 cells was evaluated using the cell membrane permeable fluorescent probe HPF to detect Type I ROS following treatment with V‐LNP formulations and light activation. The Type I ROS generation profile inside cells mirrored the cell‐free assay results, with Lipo 20:0 BPD‐PC producing the lowest Type I ROS and LNP BPD‐Cholesterol producing the highest Type I ROS. These findings confirm that the photochemical ROS patterns of the V‐LNPs are maintained after cellular internalization.

### 2.4 Determining Subcellular Localization of the V‐LNPs (Input Metric for PCA)

The subcellular localization of photosensitizers can impact PDT efficacy, and even cell death modality, by selectively damaging certain organelles or cellular compartments. Direct ER photodamage has been suggested to be a key regulator of photochemical ICD, with hypericin, an ER‐localizing photosensitizer, being one of the first sensitizers studied in detail in the context of ICD.^[^ ^38^, ^39^, ^74^ ^]^ However, a number of other photosensitizers have been used for photochemical ICD induction even though these localize in the lysosomes,^[^ ^1^, ^24^, ^33^, ^36^ ^]^ mitochondria,^[^ ^38^, ^75^ ^]^ Golgi Apparatus,^[^ ^76^ ^]^ or combinations of multiple organelles.^[^ ^28^, ^36^, ^76^ ^]^ As such, it remains unclear what impact photosensitizer subcellular localization has on the efficiency of photochemical ICD induction in cancer cells, and especially so for LNP formulations of photosensitizers which remain understudied.

**Figure 2:**
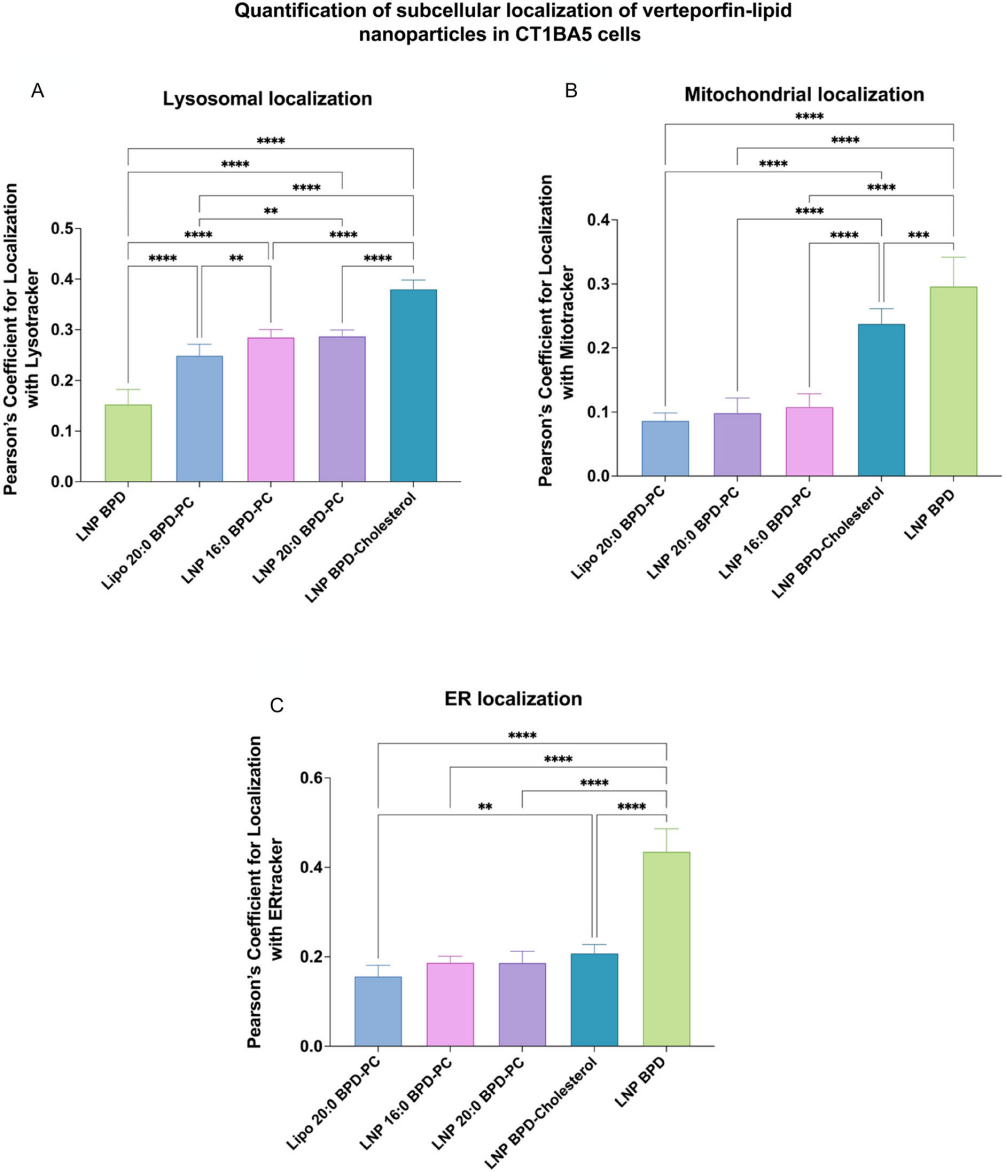
Pearson's coefficient values for colocalization of V‐LNPs with markers for A) lysosomes, B) mitochondria, and C) ER in CT1BA5 cells. (All data are presented as mean ± S.D., (*n* = 6). Statistical significance was calculated using a one‐way ANOVA test on GraphPad Prism v10.4.1, *: *P* < 0.1, **: *P* < 0.01, ***: *P* < 0.001, ****: *P* < 0.0001).

We therefore quantified the intracellular localization of V‐LNPs in CT1BA5 cells by determining Pearson's coefficient values for colocalization with respective organelle markers using confocal microscopy. These Pearson's coefficient values served as input metrics for the subsequent PCA analysis to determine the association of V‐LNP subcellular localization with exposure of ICD markers. As shown in **Figure** **2**, S17, S19, and S21, Supporting Information, Lipo 20:0 BPD‐PC, LNP 20:0 BPD‐PC, and LNP 16:0 BPD‐PC exhibited predominant localization within lysosomes. LNP BPD‐Cholesterol on the other hand localized in both the lysosomes and the mitochondria. LNP BPD exhibited the greatest accumulation in both the ER and mitochondrial. The quantification of the Pearson's coefficient values was used for the upcoming PCA analysis to determine not only the impact of the site(s) of subcellular localization that was important for photochemical ICD, but also the impact of the degree of accumulation in the respective organelles.

### 2.5 Measuring the Phototoxicity of V‐LNPs (Input Condition for PCA)

We investigated the effects of light activation of the V‐LNPs on the metabolic activity of CT1BA5 and 6620c1 pancreatic cancer cells. As shown in Figure S7A, Supporting Information, all V‐LNP formulations (other than Lipo 20:0 BPD‐PC) exhibited mild dark toxicity in CT1BA5 cells at high concentrations in the 10 μM range, which may be attributed to the ionizable lipid SM‐102.^[^ ^77^ ^]^ All V‐LNPs exhibited nanomolar activity in CT1BA5 and 6620c1 cells following activation with 20 J cm^−2^ of 690 nm light at 18 mW cm^−2^ (Figure S7B,D, Supporting Information, and **Table** **2**). LNP BPD and LNP BPD‐Cholesterol were the most phototoxic, followed by LNP 20:0 BPD‐PC, LNP 16:0 BPD‐PC, and then Lipo 20:0 BPD‐PC. The IC_25_, IC_50_, and IC_75_ values for each formulation were extrapolated from the cell viability curve which was obtained using an MTT assay. Cellular uptake levels (Input metric for PCA) coincided with phototoxicity levels, with LNP BPD‐Cholesterol being most efficient at 2.41 **× **10^−7^ nmol BPD‐Cholesterol per cell and Lipo 20:0 BPD‐PC being least efficient at 5.80 **× **10^−8^ nmol 20:0 BPD‐PC per cell (Figure S7C, Supporting Information).

**Table 2:** Summary of verteporfin‐equivalent inhibitory concentrations of the panel of V‐LNPs in CT1BA5 cells activated with 690 nm light using a fluence of 20 J cm^−2^ and an irradiance of 18 mW cm^−2^. Results were obtained from the MTT assay. Values are mean, *n* = 6 +/− SD.

V‐LNP	IC_25_ [nM ± S.D.]	IC_50_ [nM ± S.D.]	IC_75_ [nM ± S.D.]
Lipo 20:0 BPD‐PCa)	18.8 ± 2.8	39.9 ± 3.3	77.8 ± 6.5
LNP 20:0 BPD‐PCa)	11.4 ± 1.9	26.2 ± 2.1	49.7 ± 3.6
LNP 16:0 BPD‐PC	25.3 ± 3.8	49.0 ± 4.3	90.8 ± 12.7
LNP BPD	7.2 ± 0.9	13.9 ± 2.9	27.1 ± 4.2
LNP BPD‐Cholesterol	5.9 ± 2.1	14.8 ± 2.6	30.3 ± 3.2

a) Data were adapted from our recently published study.^[^ ^29^ ^]^

### 2.6 Measuring Photochemically‐Induced Exposure of ICD Markers (Output Metrics for PCA)

**Figure 3:**
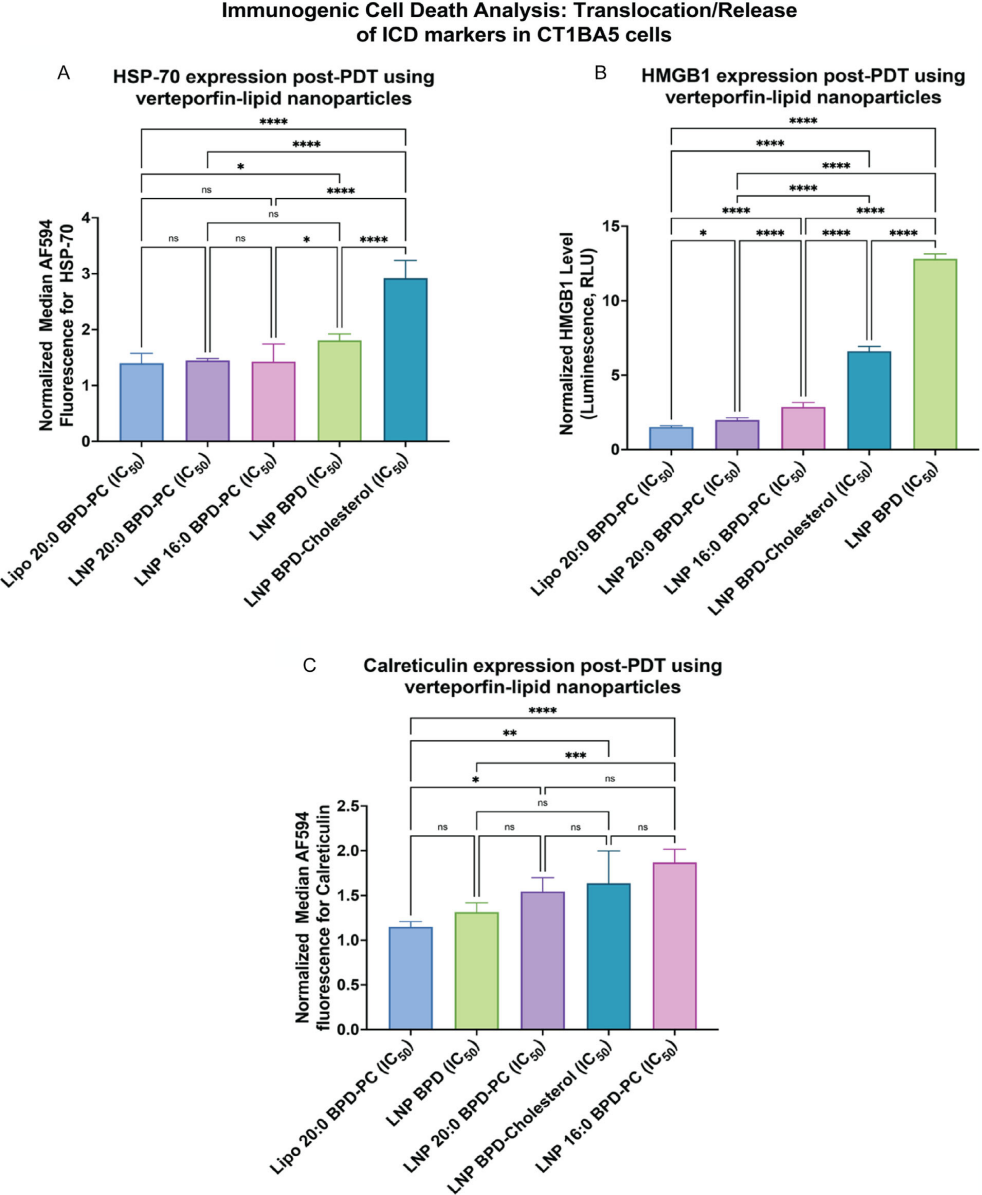
Exposure of the ICD markers A) HSP‐70, B) HMGB1, and C) calreticulin in CT1BA5 cells following 690 nm light activation using a fluence of 20 J cm^−2^ and IC_50_ verteporfin‐equivalent concentration of V‐LNPs. (All data was normalized to untreated cells and are presented as mean ± S.D., (*n* = 6). Statistical significance was calculated using a one‐way ANOVA test on GraphPad Prism v10.4.1, *: *P* < 0.1, **: *P* < 0.01, ***: *P* < 0.001, ****: *P* < 0.0001).

ICD can be induced by a variety of treatment modalities to enhance antitumor immunity.^[^ ^78^ ^]^ Conventional chemotherapeutics, such as anthracyclines and oxaliplatin, as well as radiation, and immune adjuvants, are known to induce ICD.^[^ ^79^ ^]^ Novel ICD inducers are currently in preclinical and clinical development, and have been shown to augment immunotherapy responses. Examples include PT‐112, a platinum‐pyrophosphate conjugate that synergizes with PD‐1 or PD‐L1 blockade^[^ ^80^ ^]^ and the oncolytic peptide LTX‐315, which overcomes resistance to CTLA‐4 blockade.^[^ ^81^ ^]^ Recent advances also include nanoparticle‐based platforms and engineered nanovaccines designed to amplify ICD signatures, such as calreticulin exposure, ATP release, and HMGB1 secretion.^[^ ^78^, ^82^ ^]^ As mentioned earlier, photochemical stimulation is one of the most effective inducers of ICD^[^ ^11^ ^]^ and offers the added advantage of focal spatiotemporal control over ICD induction, which systemically administered ICD inducers are not able to offer. Photochemical ICD results in the release/translocation of DAMPs such as HSP‐70,^[^ ^83^, ^84^ ^]^ calreticulin,^[^ ^21^, ^22^, ^28^ ^]^ and HMGB1^[^ ^22^, ^28^ ^]^ in dying or stressed cells.^[^ ^4^, ^11^ ^]^ In vivo, these DAMPs can activate macrophages and DCs, which further activate T‐cell mediated adaptive immunity through tumor‐specific antigens.^[^ ^22^, ^31^ ^]^ We and others have shown that various verteporfin‐based lipid nanoformulations can elevate ICD marker exposure in vitro and in vivo, and that solid LNPs may be more efficient than liposomes.^[^ ^24^, ^28^, ^29^, ^30^ ^]^ In this study, we quantified the exposure of the ICD markers HSP‐70, HMGB1, and calreticulin following activation of the V‐LNPs with verteporfin‐equivalent IC_25_, IC_50_, and IC_75_ concentrations in CT1BA5 cells. This data was later used in the PCA analysis as the output metrics. **Figure** **3** shows the exposure of HSP‐70, HMGB1, and calreticulin using the IC_50_ verteporfin‐equivalent concentration of V‐LNPs. LNP BPD‐Cholesterol exhibited the greatest exposure of HSP‐70, followed by LNP BPD, while LNP 20:0 BPD‐PC and Lipo 20:0 BPD‐PC exhibited the lowest exposure of HSP‐70. Similar trends were observed for HMGB1 exposure. LNP BPD‐Cholesterol and LNP 16:0 BPD‐PC exhibited the greatest exposure of calreticulin, while Lipo 20:0 BPD‐PC was lowest. V‐LNPs that induce the highest levels of phototoxicity are most strongly associated with ICD marker exposure. Data for all three ICD markers with IC_25_, IC_50_, and IC_75_ concentrations of V‐LNPs are presented in Figure S8–S10, Supporting Information. We also validated the efficiency of ICD marker exposure following activation of V‐LNPs in 6620c1 cells at IC_50_ concentrations. Similar trends were observed as with CT1BA5 cells, demonstrating that LNP BPD‐Cholesterol and LNP BPD were most efficient at elevating HMGB1 and HSP‐70 exposure (Figure S14, Supporting Information).

To verify that ICD marker exposure can ultimately lead to immune cell activation, we cocultured mouse spleen‐derived DCs (CD11c^+^) with CT1BA5 cells subjected to photochemical activation of LNP BPD‐Cholesterol at the IC_50_ BPD‐equivalent concentration. DCs were cocultured with untreated CT1BA5 cells as a control. DCs cocultured with CT1BA5 cells undergoing photochemical ICD exhibited a 48% increase in CD80 expression and a 20% increase in CD86 expression, with respect to DCs cocultured with untreated control CT1BA5 cells (Flow cytometry, Figure S16A,B, Supporting Information). Elevations in both CD80 and CD86 are indicative of DC activation, which is the critical next step in the immune activation cycle that can ultimately lead to T‐cell mediated responses.

### 2.7 2D Correlations of V‐LNPs Attributes and Exposure of ICD Markers

**Figure 4:**
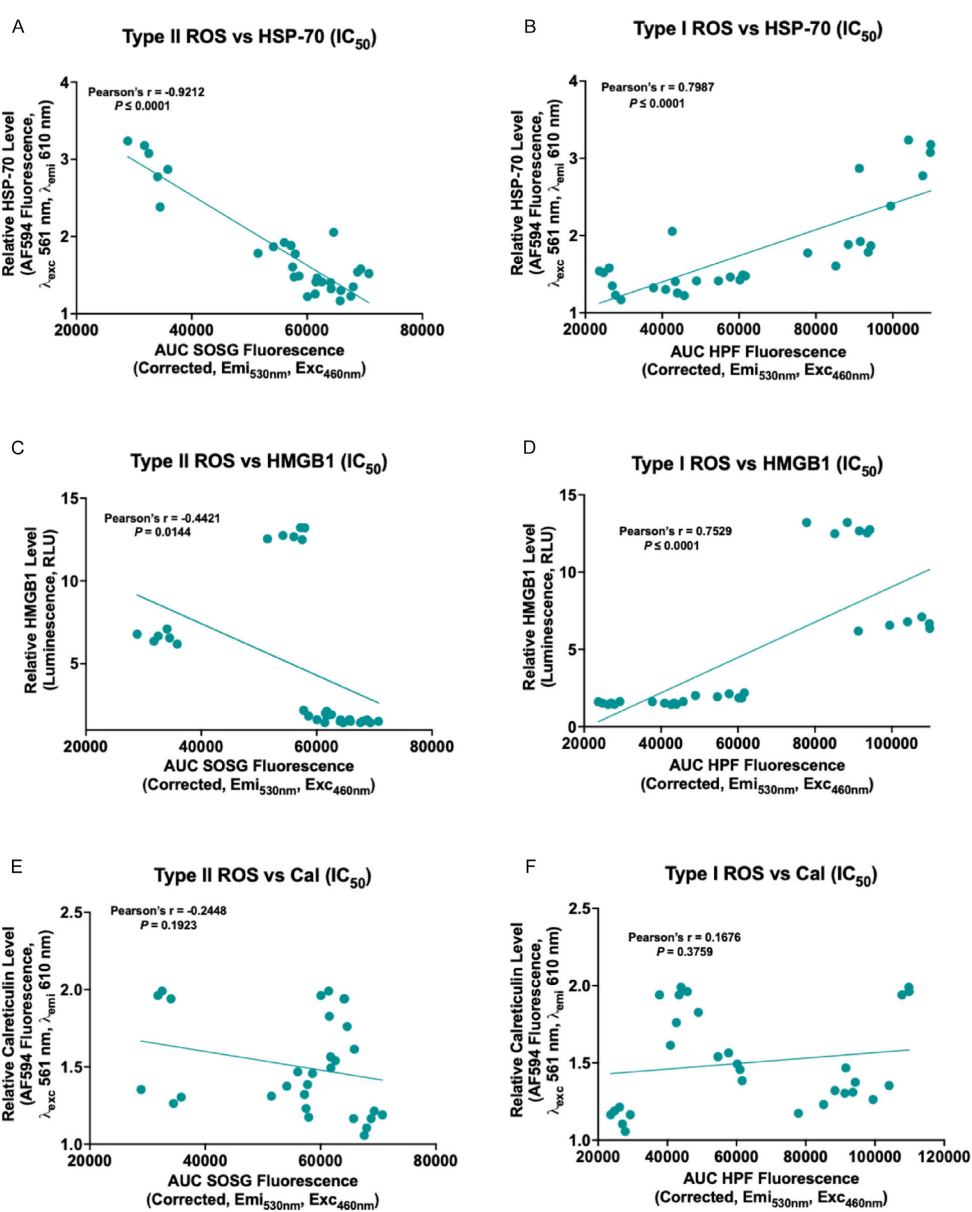
Representative scatter plots showing the relationships between the exposure of the ICD marker HSP‐70, HMGB‐1, and calreticulin (Cal) at IC_50_ verteporfin‐equivalent concentrations of V‐LNPs in CT1BA5 cells and Type II ROS A,C,E) or Type I ROS B,D,F). HSP‐70 and calreticulin were measured using flow cytometry and HMGB1 was measured using a bioluminescence kit (Promega). Type II ROS is represented by the AUC of the emission curves of the SOSG probe and Type I ROS is represented by the AUC of the emission curves of the HPF probe following 690 nm activation of V‐LNPs. The correlation analysis was conducted using GraphPad Prism v10.4.1.

As a preliminary 2D analysis prior to the full PCA analysis, we explored whether correlations existed individually between different V‐LNPs attributes and the exposure of ICD markers using IC_25_, IC_50_, and IC_75_ verteporfin‐equivalent concentrations of V‐LNPs in CT1BA5 cells. **Figure** **4** shows representative scatter plots of the relationships between the type of ROS generated and ICD marker exposure using IC_50_ verteporfin‐equivalent concentrations of V‐LNPs. For both HSP‐70 and HMGB1 exposure, statistically significant positive correlations were observed with Type I ROS and statistically significant negative correlations were observed with Type II ROS. The impact of the type of ROS generated and exposure of calreticulin was mixed. Similar trends were observed for IC_25_ and IC_75_, and the exposure of HSP‐70 and HMGB1 was more pronounced as the verteporfin‐equivalent concentrations of V‐LNPs increased (Figure S11–S13, Supporting Information). While Type I ROS production was strongly associated with HSP70 and HMGB1 exposure, calreticulin did not follow the same trend—reflecting differences in the regulation and timing of DAMP exposure. Calreticulin translocation is an early, preapoptotic event that is tightly regulated by ER stress signaling pathways such as PERK–eIF2α.^[^ ^85^ ^]^ Its surface exposure can be dose‐dependent or biphasic^[^ ^86^ ^]^ and is influenced by upstream processes like ER‐to‐membrane trafficking and eIF2α phosphorylation.^[^ ^87^ ^]^ In contrast, HSP70 and HMGB1 are typically released during later stages of stress or membrane rupture and appear to respond more broadly to oxidative damage caused by Type I ROS. These distinctions help explain why certain forms of ICD often lack robust calreticulin exposure.^[^ ^15^ ^]^ Overall, these findings highlight that although Type I ROS is a key driver of ICD, the specific DAMPs exposed are shaped by the localization of ROS and the temporal dynamics of cell death.

To further confirm the relationship between the exposure of ICD markers and the type of ROS production by V‐LNPs, we performed a correlation analysis using 6620c1 cells. Figure S15, Supporting Information, shows a representative scatter plot illustrating the relationship between ICD marker exposure in 6620c1 cells and the type of ROS using IC_50_ verteporfin‐equivalent concentrations of the V‐LNPs. A statistically significant negative correlation was observed between Type II ROS and the exposure of ICD markers HSP‐70 and HMGB1 using IC_50_ verteporfin‐equivalent concentrations. Furthermore, a statistically significant positive correlation was observed between Type I ROS and the exposure of ICD markers HSP‐70 and HMGB1. These findings are consistent with our observations in CT1BA5 cells.

Additionally, we investigated the relationship between the subcellular localization of V‐LNPs and the exposure of ICD markers. As shown in Figure S18, Supporting Information, no statistically significant correlation was observed between localization of V‐LNPs in lysosomes and the exposure of ICD markers. However, a statistically significant positive correlation between mitochondrial localization and ER localization of V‐LNPs with HMGB1 and HSP70 exposure was found (Figure S20 and S22, Supporting Information). A statistically significant positive correlation between mitochondrial localization and ER localization with calreticulin exposure was found only at IC_75_ concentrations (Figure S20 and S22, Supporting Information).

**Figure 5:**
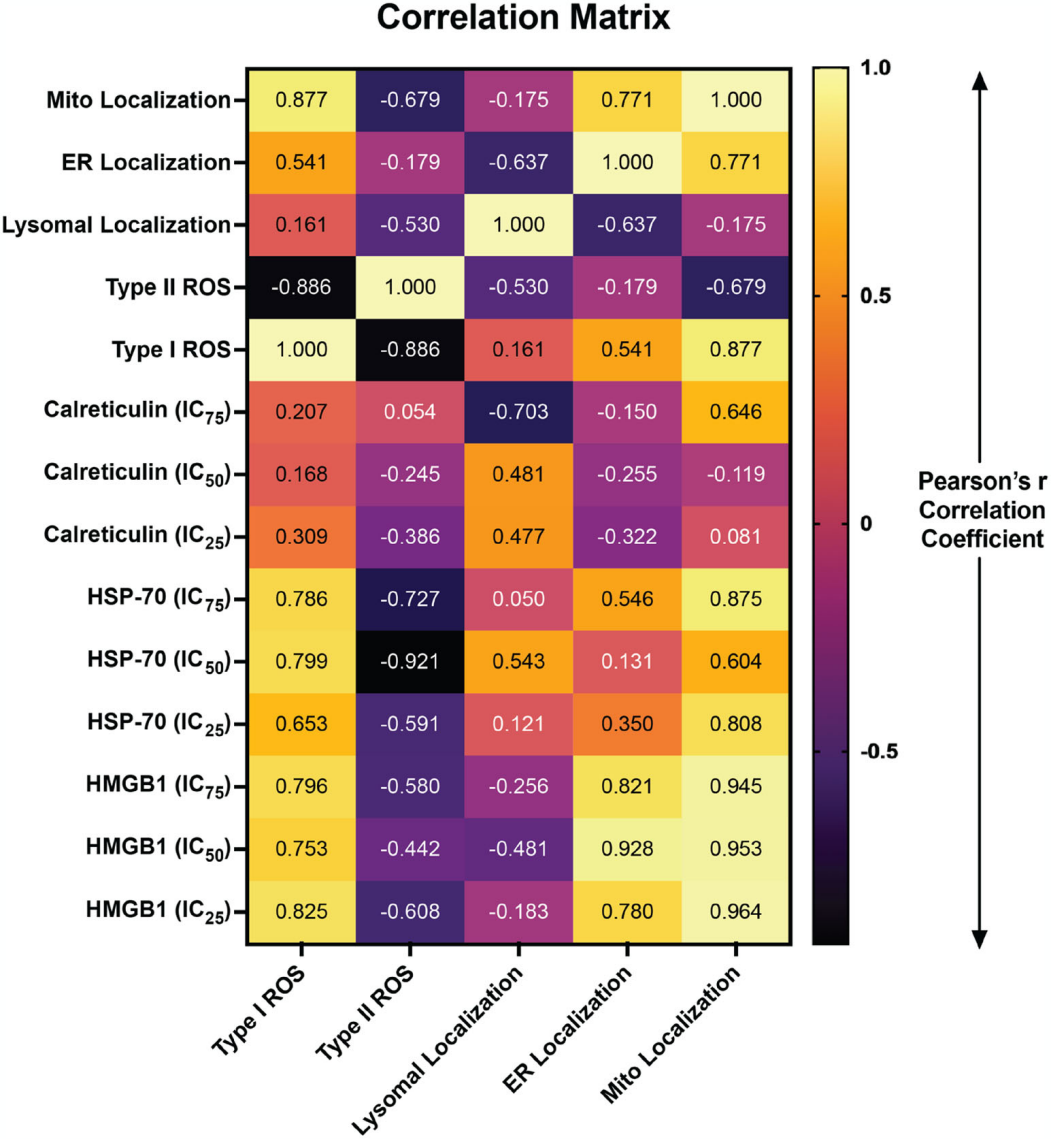
Heatmap showing the Pearson's coefficient matrix of the relationships between ROS generation (Type I and Type II), ICD markers at different levels of phototoxicity (calreticulin, HSP‐70, and HMGB1), and subcellular localization (lysosomes, mitochondria (Mito), and ER) for all the V‐LNPs. The analysis was conducted using GraphPad Prism v10.4.1.

The relationships between the type of ROS species produced, subcellular localization, and photochemical ICD marker exposure using IC_25_, IC_50_, and IC_75_ doses of V‐LNPs are summarized in the correlation matrix heat map of Pearson's coefficients in **Figure** **5**. The strongest positive correlations were observed between Type I ROS and the exposure of HSP‐70, Type I ROS, and the exposure of HMGB1, and mitochondrial localization and the exposure of HSP‐70 and HMGB1. ER localization of V‐LNPs also appeared to be correlated with the exposure of HMGB1 and, to a lesser extent, HSP‐70.

### 2.8 PCA to Determine the Multivariate Relationships Between the V‐LNP Attributes and Exposure of ICD Markers

PCA was performed to evaluate the collective contribution of multiple V‐LNP attributes on the efficiency of ICD marker exposure following light activation under various treatment conditions. We first determined the contribution of different principal components (PCs) to the overall variance in the dataset.^[^ ^88^ ^]^ The dataset included the various V‐LNP attributes and ICD marker quantitation at IC_25_, IC_50_, and IC_75_ (loadings). Figure S23, Supporting Information, illustrates the proportion of variance explained by each PC, both individually and cumulatively. The first two PCs (PC1 and PC2) were selected based on their cumulative energies, which accounted for 80.65% of the total energies. Individually, PC1 and PC2 accounted for 56.70% and 23.95% of the total energies, respectively. Subsequent components contributed less, and the decline in energy contribution after PC2 supports our selection of PC1 and PC2, which capture the most relevant data structure.

**Figure 6:**
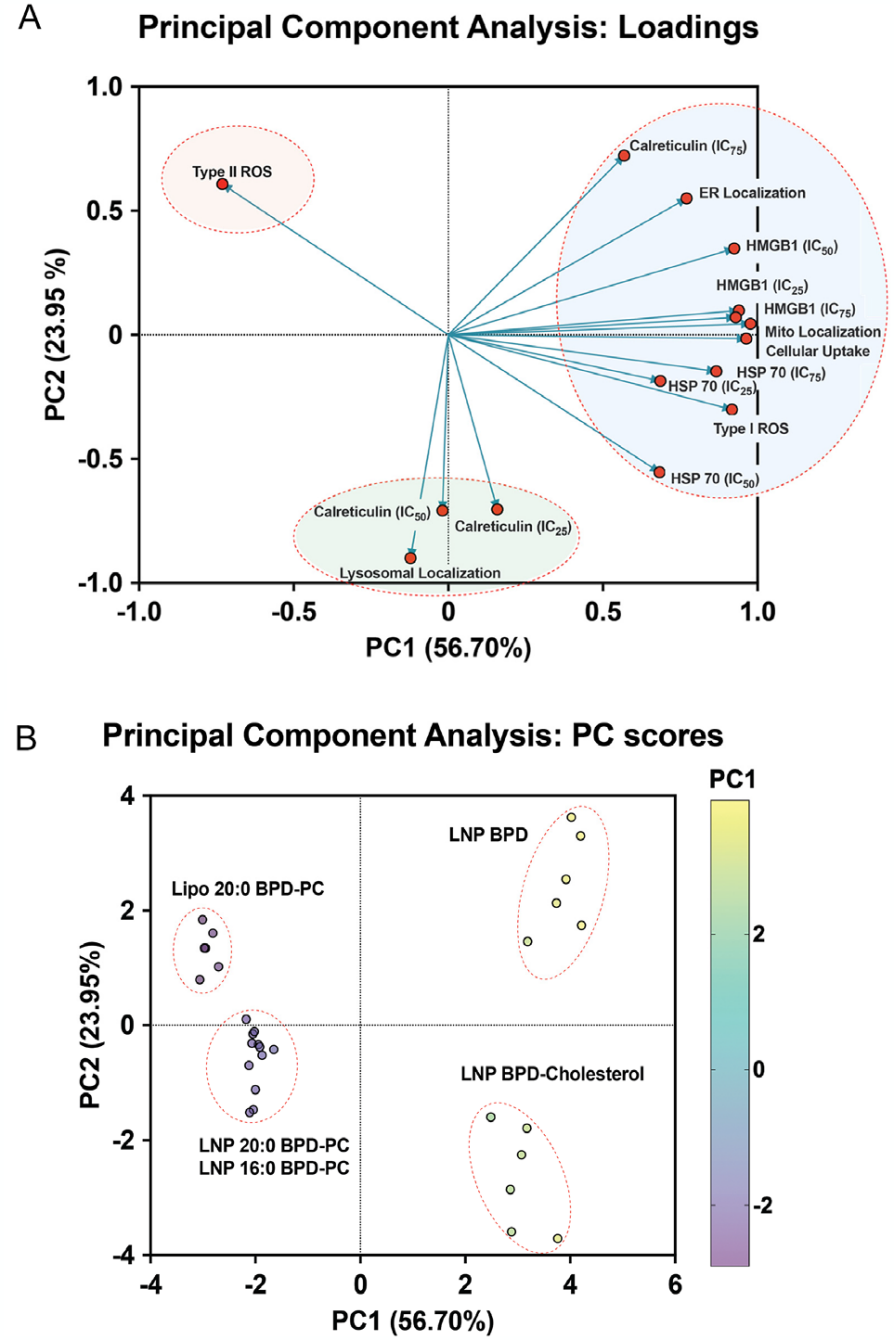
PCA of various V‐LNP attributes and treatment conditions leading to photochemical ICD. A) PCA loading plot illustrating the contribution of individual variables to each principal component, indicating important components driving variability in the dataset. B) PC score plot showing sample distribution along the principal components, highlighting clustering patterns and group separations. PCA analysis was conducted using GraphPad Prism v10.4.1.

As shown in **Figure** **6**A, the loading plot illustrates the contribution of each variable (V‐LNP attributes and ICD marker exposure) to PC1 and PC2. This loading plot identifies the relationships between the variables, where positively correlated variables are positioned closely together and negatively correlated variables are positioned further apart. We observed three distinct clusters across both the components (PC1 and PC2). The first cluster on the PCA loadings plot included only Type II ROS, with no other variables, including ICD marker exposure, associated with this particular V‐LNP attribute. This suggests that V‐LNPs that produce greater quantities of Type II ROS are not conducive to the exposure of any ICD markers. The second cluster on the PCA loadings plot included all of Type I ROS, cellular uptake efficiency, mitochondrial, and ER localization, the exposure of HMGB1 and HSP‐70 at all verteporfin‐equivalent concentrations of V‐LNPs (IC_25_, IC_50_, and IC_75_), and the exposure of calreticulin with IC_75_ conditions. This suggests that V‐LNPs that produce greater quantities of Type I ROS, improved cellular uptake efficiency, and also localize in the mitochondria and/or the ER, are more closely associated with the exposure ICD markers HMGB1 and HSP‐70, and only at high doses of photochemical stimulation, are associated with the exposure of calreticulin. The third cluster on the PCA loadings plot included lysosomal localization and calreticulin exposure only at IC_25_ and IC_50_ verteporfin‐equivalent concentrations of V‐LNPs. This suggests that V‐LNPs that localize in lysosomes are associated with the exposure of calreticulin, but only at lower doses of photochemical stimulation. Calreticulin exposure, being an early preapoptotic event, did not follow the same trend, which is likely due to its tight regulation by ER stress pathways that govern its translocation to the cell surface.^[^ ^85^ ^]^ In contrast, exposure or release of HSP‐70 and HMGB1 occurs during late apoptosis or necrosis, reflecting significant cellular stress and membrane permeabilization.^[^ ^89^, ^90^ ^]^ Overall, the results of the PCA analysis suggest that V‐LNPs must be designed to generate the highest amount of Type I ROS, the most efficient cellular uptake, and accumulate in both the mitochondria and ER, in order to induce the greatest amount of ICD marker exposure. It is important to note also, that our results suggest that the greater the degree of V‐LNPs phototoxicity, the greater the efficiency of ICD marker exposure for a given V‐LNP, with ICD marker exposure detected even at partially phototoxic IC_25_ doses.

Using PCA analysis, we were also able to cluster the V‐LNPs themselves based on their collective features, namely their attributes and their efficiencies of ICD marker exposure. We used PCA analysis to assign PC scores to all the V‐LNPs. PC scores are numerical values that define the positioning of each sample along the PCs, offering a simplified representation of the complex dataset for the V‐LNP features. V‐LNPs with similar PC scores cluster together, suggesting they have similarities in their collective features. As shown in Figure 6B, distinct clustering patterns were observed: LNP BPD and LNP BPD‐Cholesterol clustered on the positive side of PC1, suggesting that they exhibit the greatest similarities in their collective features. LNP 20:0 BPD‐PC and LNP 16:0 BPD‐PC clustered together at negative PC1 and near zero PC2, suggesting similarities in their collective features, which are significantly different from the collective features of LNP BPD and LNP BPD‐Cholesterol. Lipo 20:0 BPD‐PC clustered alone toward the positive PC2 component, suggesting that their collective features are distinct from the remaining V‐LNPs. This pattern also suggests that the choice of lipid formulation vehicle (liposomes or solid LNPs) impacts their collective features more when using 20:0 BPD‐PC specifically, which is consistent with some of our preliminary published work on 20:0 BPD‐PC formulations.^[^ ^29^ ^]^ The clustering of LNP BPD‐Cholesterol and LNP BPD in the positive PC1 region coincides with their greater efficiency in producing Type I ROS, higher cellular uptake efficiencies, mitochondrial and ER localization, and exposure of HSP‐70 and HMGB1 in cancer cells following photochemical ICD.

## 3 Conclusion

Light‐activatable LNPs, among other nanoparticle preparations, have drawn significant interest as facilitators for photochemical ICD, which can be potentially used in conjunction with immune checkpoint inhibitors. In this study, we prepared a panel of V‐LNPs that exhibited marked differences in important photophysical and biological attributes, which were anticipated to contribute to photochemical ICD. These V‐LNP attributes include the type and quantity of ROS species generated following light activation, the subcellular localization in pancreatic cancer cells, and the degree of cellular phototoxicity. We used this panel of V‐LNPs to systematically study the collective attributes that are most strongly associated with the exposure of ICD markers in the pancreatic cancer cells. By quantifying these V‐LNP attributes and reducing their dimensionality of the large multivariate dataset using PCA, we identified that Type I ROS production, along with high cellular uptake efficiencies and subcellular localization in the ER and mitochondria are the most strongly associated with ICD marker exposure at IC_25_, IC_50_, and IC_75_ conditions. Conversely, and somewhat surprisingly, we found that Type II ROS production had the least association with ICD marker exposure under all conditions, and that lysosomal localization induces phototoxicity without substantial ICD marker exposure.

We report that V‐LNPs that produce more Type I ROS than Type II ROS are more efficient at inducing photochemical ICD. It is well established that Type I ROS, such as hydroxyl radicals, are significantly more reactive than singlet oxygen, with hydroxyl radical being the most powerful oxidizing agent with a redox potential of 2.34 V.^[^ ^91^ ^]^ Furthermore, Type I ROS is almost indiscriminate in its photooxidation and reacts readily with a broad spectrum of biomolecules, including DNA, proteins, and diverse membrane lipids.^[^ ^92^ ^]^ On the other hand, photooxidation by singlet oxygen is more selective and reacts predominantly with electron‐rich molecules, such as double bonds found in unsaturated fatty acids and aromatic amino acids, as well as sulfur‐containing compounds.^[^ ^93^ ^]^ This efficient and broad chemical reactivity of Type I ROS likely promotes ER and mitochondrial stress, plasma membrane rupture, and robust activation of ICD signaling cascades,^[^ ^94^ ^]^ and may explain why Type I ROS has a strong association with ICD marker exposure in our study. Furthermore, lipid components within V‐LNPs may influence ROS reactivity. Lipid components of V‐LNPs could also mediate radical‐generating chain reactions initiated by Type I ROS (including those associated with ferroptosis), thereby increasing the short half‐life of Type I ROS products and promoting oxidative stress in organelles such as the ER or mitochondria that leads to ICD.

While intriguing and of high significance, it is important to note that the associations we have identified here are for verteporfin‐based LNPs and may not hold true for other nanoformulations, such as polymeric nanoparticles, protein nanoparticles, and inorganic nanoparticle carriers. It is also important to note that these relationships were observed in vitro using 690 nm LED light with a fluence of 20 J cm^−2^ and an irradiance of 18 mW cm^−2^. However, the extent to which these associations remain consistent when using photochemical stimulation protocols with higher or lower fluences and irradiances remains unclear, as does the potential impact of irradiance gradients in vivo. To fully elucidate the role of fluence and irradiance on photochemical ICD, more comprehensive dose‐finding studies are needed, including controlled light dosimetry within solid tumors. Furthermore, the associations that we report in this study may be influenced by additional factors, such as the nature of the verteporfin conjugates that are formulated, the type of photosensitizer used, the tumor origin of the cancer cell lines, and mutations that may impact ICD marker exposure. The photochemical activation of V‐LNPs in solid tumors for the purpose of inducing ICD introduces several layers of complexity. These complexities revolve around biophysical hurdles that include heterogeneity of V‐LNP extravasation and distribution in solid 3D tumor tissue, selection of drug‐light intervals, varying photochemical reactions at different tissue oxygenation levels, differential gradients of light fluence and irradiance through solid tumors, and variables introduced by the choice of light delivery (for example external beam or interstitial fiber light activation), among several other factors. With respect to V‐LNP penetration in 3D tumor tissue in vivo, heterogenous distribution throughout the tumors will likely impact the extent of tissue photodamage. If durable immune responses are elicited as a result of photochemical ICD by V‐LNPs, residual untreated tumor tissue that escaped direct photodamage may be immunologically cleared. However, this is not fully known at this point and will require optimization of V‐LNP and light dose parameters in upcoming preclinical animal studies. Regarding ROS production by V‐LNPs, we have shown that V‐LNPs produce differential Type I and Type II ROS under normoxic conditions in vitro. It is not known how oxygenation gradients in tumors in vivo will impact the amount and type of ROS produced by V‐LNPs, and how that will effect ICD induction in vivo is also unknown. Regarding vascular effects, vascular targeted PDT has been reported to elicit potent immune responses that can augment immune checkpoint therapy.^[^ ^95^, ^96^ ^]^ It is not known to what extent vascular photodamage by V‐LNPs will impact immune responses in vivo and whether these effects will mask the differences in ICD that we observe in cancer cells here in vitro. These mechanisms of vascular photodynamic immune enhancement will also likely differ from those elicited by tumor cell photochemical ICD. With respect to light dosimetry, it is already known that irradiance plays a significant role in immune responses following PDT in vivo, as we have discussed earlier. How gradients of irradiance and fluence throughout tumor tissue in vivo will impact photochemical ICD by V‐LNPs will also need to be investigated in detail. It is likely that regions of high irradiance and fluence that are closest to the light source will experience tumor ablation, while distant regions will experience more of a photodynamic priming effect.^[^ ^8^ ^]^ At a cellular level, we show that photochemical ICD by V‐LNPs can provoke ICD marker exposure at mildly phototoxic doses (IC_25_) and increasingly so at higher doses (IC_50_ and IC_75_). We have previously shown that simple liposomal formulations of verteporfin can induce photochemical ICD in vivo mouse tumor models of head and neck cancer using external beam photoactivation,^[^ ^28^ ^]^ but we have not explored the spatial distribution of ICD marker exposure with respect to the angle of incident light. This will be even more important when considering the light delivery technique and how that impacts photochemical ICD across tumor cross‐sections.

In certain tissues, light penetration may be more limited and may prohibit photochemical ICD using the V‐LNPs we present here.^[^ ^97^ ^]^ Sonodynamic therapy (SDT) is an emerging alternative that enables deeper tissue activation of compounds including photosensitizers by ultrasound waves. SDT addresses the limitations of light penetration, although the ROS yield is often lower than with photochemical activation, and dosimetry is poorly understood.^[^ ^98^ ^]^ Another alternative energy source that has been used to activate photosensitizers in preclinical and clinical trials is X‐rays for radiodynamic therapy. It is worth noting that the results presented in this study are specific to the activation of V‐LNPs with 690 nm light, and further exploration is needed to determine if ICD induction can be achieved with V‐LNPs using these alternative excitation modalities. Chemodynamic therapy is also another emerging approach that complements PDT and leverages the tumor microenvironment for on‐demand ROS production without external stimuli, but its reliance on endogenous substrates and its risk of off‐target effects requires careful consideration.^[^ ^99^ ^]^

Future work will also investigate how the efficiency of photochemical ICD relates to the mechanisms of cell death, including pyroptosis, ferroptosis, and immunogenic necroptosis, in multiple tumor cell types. We will examine key markers of ER and mitochondrial stress, including CHOP, calnexin, cytochrome c, and JC‐1, to evaluate the mechanisms underlying organelle‐specific damage and stress responses that contribute to ICD. These studies will provide further insights into why there is a strong association between intracellular localization of V‐LNPs and ICD marker exposure. We will also investigate the emerging roles of photodamage‐induced calcium flux and lipid peroxidation in the initiation of ICD using V‐LNPs, especially in relation to ferroptosis.^[^ ^100^ ^]^ Future work will also explore the relationship between the associations we report here for V‐LNPs and with antitumor immune responses in mouse models of PDAC that exhibit immunologic heterogeneity. These studies will be critical to determine whether enhanced ICD marker exposure and increased DC activation translate into robust T‐cell activation, and long‐term antitumor immunity in vivo, especially in immunosuppressed PDAC models. In these upcoming in vivo studies, we will directly compare two V‐LNPs that represent the extremes amongst our panel: Lipo 20:0 BPD‐PC, which is least efficient at photochemical ICD marker exposure in vitro, and LNP BPD‐Cholesterol which is most efficient. Both formulations will be tested alone and in combination with an immune checkpoint inhibitor (for example, anti‐PD‐L1 antibodies) to assess potential synergistic effects of T‐cell mediated responses. Key in vivo endpoints will include tumor growth inhibition and tumor rechallenge studies, animal survival, and spatial distribution of ICD markers (e.g., HSP‐70, calreticulin, HMGB1) and tumor‐infiltrating CD8^+^ T cells with respect to V‐LNP distribution and incident light propagation in tumors. These upcoming in vivo studies will demonstrate to what extent in vitro data‐driven predictions of photochemical ICD by V‐LNPs translate to durable immune‐mediated antitumor responses.

The findings that we present in this data‐driven study provide important insights into the multiparametric mechanisms underlying photochemical ICD induced by V‐LNPs. This mechanistic understanding can provide a framework for the rational design of new verteporfin‐lipid‐based nanoparticle regimens with the goal of fine‐tuning ICD in pursuit of enhancing immune responses in difficult‐to‐treat cancers.

## Supporting Information

Supporting Information is available from the Wiley Online Library or from the author.

## Conflict of Interest

The authors declare no conflict of interest.

## Supporting information

Supplementary Material

## Data Availability

The data that support the findings of this study are available in the supplementary material of this article.
