# Supplementary material for: Fine‐Tuning Photochemical Immunogenic Cell Death by a Panel of Verteporfin‐Lipid Nanoparticles: A Data‐Driven Approach
Source: Small Sci. 2025 Oct 5;5(11):2500290. doi: 10.1002/smsc.202500290 (PMC12622458; doi:10.1002/smsc.202500290)
Supplement: Supplementary file 1 — Supplementary Material [file SMSC-5-2500290-s001.pdf]

Supplementary Data

Fine-tuning PhotochemicalImmunogenic Cell Death by a Panel of Verteporfin-Lipid Nanoparticles: A Data-driven Approach

Nimit Shah[a], Maxwell Bortei Quaye[a], Siddharth Reddy Soma[a], Meghana Sree Vadlamudi[a],Doha Mahmoud[a], Ashritha Malkoochi[a], Taksheel Rao Aileni[a], Chanda Bhandari[a],Kunal Karambelkar[a], Tayyaba Hasan[b,c], Mladen Korbelik[d], Baowei Fei[a,e], Xinxin Song[f] and Girgis Obaid[a]*

[a] Department of Bioengineering, The University of Texas at Dallas, Richardson, TX 75080, United States
[b]Wellman Center for Photomedicine, Massachusetts General Hospital and Harvard Medical School, Boston, Massachusetts, 02114, United States
[c]Division of Health Sciences and Technology, Harvard University and Massachusetts Institute of Technology, Cambridge, Massachusetts, 02139, United States
[d] Department of Integrative Oncology, BC Cancer, Vancouver, BC, V5Z 1L3, Canada
[e]Department of Radiology, UT Southwestern Medical Center, Dallas, TX 75390, United States
[f] Department of Surgery, UT Southwestern Medical Center, Dallas, TX 75390, United States

KEYWORDS: benzoporphyrin derivative |correlations | immunogenic cell death | liposomes | photodynamic therapy | principal component analysis | reactive oxygen species | solid lipid nanoparticles | subcellular localization

Material and methods

Synthesis of 16:0 BPD-PC, 20:0 BPD-PC, and BPD-Cholesterol: Verteporfin, also known as benzoporphyrin derivative (BPD, US Pharmacopeia), was conjugatedto 1-arachidoyl-2-hydroxy-*sn*-glycero-3-phosphocholine (20:0 lyso PC, Avanti), 1-palmitoyl-2-hydroxy-*sn*-glycero-3-phosphocholine (16:0 lyso PC,Avanti) and cholesterol through Steglich esterification using a previously described method.[1–3] Briefly, lipids (16:0 lyso PC, 20:0 lyso PC, or cholesterol), BPD, 1-ethyl-3-(3-dimethylaminopropyl) carbodiimide (EDC; Sigma-Aldrich), 4-(Dimethylamino) pyridine (DMAP, Sigma-Aldrich), and N,N-Diisopropylethylamine (DIPEA, Sigma-Aldrich)were mixed at molar ratios of 1:5:50:25:60, respectively, in 5 mL of dichloromethane (DCM; Fischer Scientific, high-performance liquid chromatography (HPLC) grade) and stirred at 2500 RPM on a magnetic stirrer for 72 h at room temperature to prepare16:0 BPD-PC, 20:0 BPD-PC, and BPD-Cholesterol, respectively. 16:0 BPD-PC, 20:0 BPD-PC, and BPD-Cholesterolwere purified using preparatory thin-layer chromatography and extracted in a 2:1 dichloromethane/methanol mixture. The extracted 16:0 BPD-PC, 20:0 BPD-PC, and BPD-Cholesterolwere then filtered using a 0.22 µm polytetrafluoroethylene (PTFE) filter and stored in chloroform at −20 °C in the dark.

Synthesis of Lipo 20:0 BPD-PC: Liposomal (Lipo) 20:0 BPD-PC was preparedusing our previously publishedmethod.[1,4,5] Briefly the lipids, 2-Dipalmitoyl-sn-glycero-3-phosphocholine (DPPC, Avanti), cholesterol,1,2-Distearoyl-sn-Glycero-3-Phosphoethanolamine with conjugated methoxy-poly(ethylene glycol) (DSPE-mPEG$_{2000}$, Avanti), and 20:0 BPD-PC were mixed in chloroform at a molar ratio of 0.675:0.300:0.015:0.010, respectively. Lipo 20:0 BPD-PC was prepared by theconventional thin-film hydration method by hydrating the thin-film of lipids with Dulbecco's Phosphate Buffered Saline (DPBS; no Calcium, no Magnesium; Corning) followed by ultrasonication (Ultrasonic probe sonicator; Fisher Scientific) for a total of 30 min (20 s on/40 s off cycles) at 42 °C in the dark. Lipo 20:0 BPD-PC was stored at 4°C in the dark.

Synthesis of LNP BPD, LNP 16:0 BPD-PC, LNP 20:0 BPD-PC, and LNP BPD-Cholesterol: All solid lipid nanoparticle (LNP) formulations ofunmodified BPD, 16:0 BPD-PC, 20:0 BPD-PC, and BPD-Cholesterolwere prepared using adaptations of our previously published method.[6] Briefly, all the lipid components, including 1,2-Dipalmitoyl-sn-glycero-3-phosphocholine (DPPC, Avanti), 1,2-Dimyristoyl-sn-glycero-3-phosphocholine (DMG-PEG$_{2000}$, NOF America Corporation), cholesterol, the ionizable lipid 1-octylnonyl 8-[(2-hydroxyethyl)[6-oxo-6-(undecyloxy)hexyl]amino]-octanoate (SM-102, BroadPharm) and the photosensitizers (unmodified BPD, 16:0 BPD-PC, 20:0 BPD-PC, or BPD-Cholesterol) were mixed at a molar ratio of 0.090:0.015:0.385:0.500:0.010, respectively.[7–9] After mixing, the lipid solutions in chloroform were dried using a stream of nitrogen gas. The resulting dried lipid film was then re-dissolved in 0.25 mL of ethanol. Using a syringe pump, the lipid solution was added dropwise using a flow rate of 0.8 mL/min into 0.75 mL of citrate buffer (pH 4.0) with continuous stirring on a magnetic stir plate at 2500 RPM. Samples were stirred for 18 h at room temperature to form the LNP BPD, LNP 16:0 BPD-PC, LNP 20:0 BPD-PC, or LNP BPD-Cholesterolformulations.The formulations werethen dialyzed using a 100 kDa dialysis tube (Spectrum Labs)against 1X DPBS (pH 7.4; no Calcium, no Magnesium; Corning) and stirred at 150 RPM. During the 24 h dialysis process, the DPBS was replaced after every 8 h. The dialyzed formulations were stored at 4 °C in the dark.

Lipo 20: BPD-PC, LNP 16:0 BPD-PC, LNP 20:0 BPD-PC, and LNP BPD-Cholesterol are collectively referred to as verteporfin-lipid nanoparticles (V-LNPs) in this study.

Physicochemical and optical characterization of V-LNPs: The hydrodynamic size and zeta potential of all the V-LNPs were measured using the Zetaziser Prodynamic light scattering (DLS) system (Malvern Panalytical Inc.). UV-Visible absorption spectrophotometry (Thermo Scientific Evolution 350) was used to measure the BPD equivalent concentration in the V-LNPs using ε_{687} nm = 34,895 M^{-1} cm^{-1} in dimethyl sulfoxide (DMSO, Sigma-Aldrich). For the absorbance spectra, 500 µL of a 5 µM BPD equivalent concentration of all the V-LNPs (LNP 20:0 BPD-PC, LNP 16:0 BPD-PC, LNP BPD-Cholesterol, LNPBPD, and Lipo 20:0 BPD-PC) weremeasured in DPBS using UV-Vis spectrophotometry. For fluorescence emission measurements, a 5 µM BPD equivalent concentration of all the V-LNPs was prepared in DPBS and serum (Fetal Bovine Serum, S11150; R&D Systems). 100 µL of the sample was placed in triplicates into a transparent bottom white well 96-plate (Corning). The fluorescence emission of each sample was then measured using a multi-plate reader (Tecan Spark plate reader) with an excitation wavelength of 435 nm and emission wavelengths ranging from 650 nm to 750 nm in 2 nm increments. The measurement settings included a manual gain of 100, 0 µs lag time, an integration time of 40 µs, and 25 flashes per read.

Cryo-Transmission electron microscopy: 3-4 µL of the V-LNP solutions were added to Lacey carbon grids (300-mesh; Ted Pella, Inc.) that were negatively glow-discharged for 80 s at 30 mA. Excess sample was removed by blotting once for 4 s with filter paper (Ted Pella, Inc.). The grids were then plunge-frozen in liquid ethane cooled by liquid nitrogen using a Leica GP2 automatic plunge freezer (Leica Microsystems). The vitrified samples were imaged using a Talos Arctica or a Glacios 200 kV cryo-transmission electron microscope (cryo-TEM) (Thermo Fisher Scientific) equipped with a K3 camera (Gatan). Serial EM software v4.1 or higher was used to collect images under low-dose conditions with a pixel size of ~1 Å/pixel. A total of 50 frames were recorded for each image over a 2.5 s exposure time with an average dose rate of ~20 e-/pixel/s.

Physical stability and photostability measurements: The physical stability of the V-LNPs at 10 µM BPD equivalent concentrations wereevaluatedunder three different conditions: at 4 °C in DPBS, at 37 °C in DPBS, at 37 °C in 10%serum-containing Dulbecco's Modified Eagle Media (DMEM) for 7 days. The hydrodynamic sizes and polydispersity indices (PDIs) were monitored daily over the course of 7 days using DLS.

5 µM BPD equivalent concentrations of the V-LNPs were prepared in DPBS or serum to evaluate photostability. 100 µL of samples was placed in a clear-bottom white walled 96-well plate (Corning). The fluorescence emission of BPD was measured after irradiation with 690 nm LED light (Biolambda) applied from the top of the plates using fluences of 0 J/cm^2, 0.5 J/cm^2, 1 J/cm^2, 1.5 J/cm^2, 2 J/cm^2, 2.5 J/cm^2, 3 J/cm^2, 3.5 J/cm^2, 4 J/cm^2, 4.5 J/cm^2, 5 J/cm^2, 10 J/cm^2, 15 J/cm^2, and 20 J/cm^2 at an irradiance of 18 mW/cm^2.

Measuring reactive oxygen species (ROS) generation: The generation of singlet oxygen was measured using the fluorometric probe Singlet Oxygen Sensor Green (SOSG, Fisher Scientific). Hydroxyl radicals and/or peroxynitrite anion were measured using the fluorometric probe hydroxyphenyl fluorescein (HPF, Fisher Scientific). For the SOSG assays, 10 µL aliquots of 50 µM SOSG wereadded to 100 µL of V-LNPs at a concentration of 5 µM BPD equivalent in DPBS. The fluorescence emission was then measured at 530 nm using an excitation wavelength of 460 nm ina multi-plate reader after irradiation with 690 nm light with increments of 0.5

J/cm^2, to a total fluence of 20 J/cm^2, at an irradiance of 18 mW/cm^2. To measure hydroxyl radicals and/or peroxynitrite anion, 20 µL aliquots of 200 µM HPF wereadded to 100 µL of V-LNPs at a concentration of 5 µM BPD equivalent in DPBS. The fluorescence emission was then measured at 530 nm using an excitation wavelength of 460 nm with a multi-plate reader after irradiation with 690 nm light with increments of 0.5 J/cm^2 to a total fluence of 20 J/cm^2 at an irradiance of 18 mW/cm^2.

Measuring intracellular ROS *in vitro*: CT1BA5 cells were seeded at a density of 50,000 cells/well in black-walled, clear-bottom 96-well plates (Corning) and incubated at 37 °C in a humidified 5% CO_2 atmosphere for 24 h. Cells were then treated with 2000 nM of each photosensitizer formulation LNP 20:0 BPD-PC, Lipo 20:0 BPD-PC, LNP BPD, LNP BPD-Cholesterol, and LNP 16:0 BPD-PC diluted in complete media and incubated for another 24 h at 37 °C. Following treatment, media was removed, and cells were incubated with 10 µM HPF(Thermo Fisher Scientific) prepared in fresh media for 30–60 min in the dark at 37 °C. HPF was added to both treatment and control groups. After HPF incubation, cells were irradiated with 690 nm light at a fluence of 20 J/cm² and an irradiance of 18 mW/cm². Following irradiation, cells were washed twice with sterile PBS (pH 7.4) and replenished with warm complete media. Fluorescence imaging was performed using an Olympus FV3000RS confocal laser scanning microscope with excitation at 488 nm and emission detection between 500–540 nm.Fluorescence intensities were analyzed using ImageJ to quantify Type I ROS.

Culture of murine pancreatic cancer cell lines: CT1BA5 cells were a kind gift from Dr. RolfBrekken at The University of Texas Southwestern Medical Center (UTSW). 6620c1 cells were a kind gift from Dr. Ben Stanger at The University of Pennsylvania. CT1BA5 cells are an isogenic pancreatic cancer cell line derived from *KPfC* (*Kras*$^{LSL\text{-}G12D}$; *Trp53*$^{fl/fl}$; *PDX*$^{Cre/+}$) mice developed by the Brekken Lab.[10] 6620c1 cells were derived from an autochthonous KPC mouse model of pancreatic adenocarcinoma by the Stanger Lab.[11,12] Both cell lines were cultured in DMEM-high glucose media supplemented with 10% fetal bovine serum (FBS) and 1X penicillin/streptomycin.

Cellular uptakeof V-LNPs: 50,000 CT1BA5 cells were seeded in a transparent bottom white walled 96-well plate (Corning) and incubated at 37 °C. After 24 h, cells were then incubated with a 250 nM BPD equivalent concentration of V-LNPs. After 24 h of incubation, cells were washed three times with 100 µL of DPBS.100 µL of DPBS containing 1% Triton X-100 was then added to each well. The plate was covered with aluminum foil and placed on a shaker for 1 h to allow complete disruption of cells and internalized V-LNPs. The fluorescence emission of the BPD variants in each well was measured using a multi-plate reader with an excitation wavelength of 435 nm and an emission wavelength of 698 nm, a gain of 150, and an integration time of 100 µs. The BPD equivalent concentration of each sample was extracted from a standard curve prepared for each formulation. The standard curves for all V-LNPs were generated by making serial range dilutions (with the highest concentration of 250 nM and the lowest concentration of 0.25 nM) of BPD equivalent concentrations of each formulation in DPBS containing 1% Triton X-100.

Determining subcellular localizationof V-LNPs: 50,000 CT1BA5 cells were seeded in a 96-well transparent glass bottom black walled plate (Celvis) and incubated at 37 °C for 24 h. After 24 h, cells were incubated with media containing V-LNPs at a BPD equivalent concentration of 2,000 nM. After 24 h incubation, the media content in each well was replaced with fresh media containing either 1 µg/ml of Hoechst (nuclei tracker; Cell

Signaling Technology), 50 nM of the Lyso-tracker (lysosome tracker; Cell Signaling Technology), 50 nM of the Mito-tracker (mitochondria tracker; Cell Signaling Technology), or 1 µM of the ER-tracker (endoplasmic reticulum tracker; Cell Signaling Technology). Cells were further incubated for 1 h in the dark at 37 °C. Cells were then washed three times using fresh media before imaging for colocalizationof the BPD variantswith the organelle markers. An Olympus FV300RS Confocal Laser Scanning Microscope was used to determine colocalization at a 100 X oil immersion objective with a 405 nm laser for Hoechst excitation, a 488 nm laser for Lysotracker excitation, a 568 nm laser for Mitotracker or ER tracker excitation, and a 647 nm laser for excitation of the BPD variants in all the V-LNPs.

Measuring cellular phototoxicity of V-LNPs: CT1BA5 and 6620c1 cells (1,500 cells per well) were seeded in clear bottom transparent 96-well plates (Corning) and incubated for 24 h at 37 °C. After 24 h, cells were incubated with culture media containing V-LNPs at 0.100 nM to 10,000 nMBPD equivalent. Following the 24h incubation, cells were irradiated using 690 nm light at a fluence of 20 J/cm^2 and an irradiance of 18 mW/cm^2. After an additional 48-h incubation, the media content from each well was replaced with 100 µL of 0.3 mg/mL of 3-(4,5-dimethylthiazol-2-yl)-2,5-diphenyltetrazolium bromide (MTT dye, Sigma-Aldrich). Cells were then incubated for at least 90 min or until visible formazan crystals formed. The contents of each well were then removed and 100 µL of DMSO was added to each well to dissolve the remaining formazan crystals. Using a multi-plate reader, the absorbance was then measured at a wavelength of 555 nm.IC_{25}, IC_{50}, and IC_{75}values for all V-LNPs were derived from non-linear regression fits using GraphPad Prism v10.4.1.

Quantifyingimmunogenic cell death (ICD) markers: CT1BA5 cells were trypsinized and seeded in a 6-well plate at a density 45,000 cells per well and then incubated at 37 °C. After 24 h incubation, the media in each well was replaced with fresh media containing IC_{25}, IC_{50}, and IC_{75}equivalent concentrations of the respective V-LNPs. After an additional24-h incubation, cells were trypsinized, transferred to Eppendorf tubes, and washed with 1X cold DPBS. To reduce non-specific binding of the antibodies used for flow cytometry, cells were pre-treatedwith 50 µL of TruStain (0.5 mg/mL, 1:5 dilution in phosphate azide buffer (PAB), BioLegend; 101320) and placed in ice for 15 mins in the dark. After incubation, to measure calreticulin and HSP-70 exposure using flow cytometry, cells were resuspended in 50 µL of anti-calreticulin antibody (0.406 µg/mL, 1:400 dilution in antibody dilution buffer, Cell Signaling Technology; clone D3E6) or 50 µL of anti-HSP-70 antibody (20 µg/mL, 1:400 dilution in antibody dilution buffer, Cell Signaling Technology; clone 4872S) and incubated for 15 min in the dark on ice. After incubation, cells were washed with PAB and resuspended in 50 µL of secondary antibody conjugated with AF594 (2 mg/mL, 1:500 dilution in PAB, Cell Signaling Technology; 8889S). Following 15 min of incubation on ice in the dark, cells were washed with PAB and then fixed with 2% formalin at room temperature. The cells were washed again with PAB and resuspended in 300 µL of PAB for flow cytometry analysis. To determine the median AF594 fluorescence emission corresponding tocell membrane calreticulin and HSP-70 levels, a 561 nm laser and a 610/20 nm bandpass detector was used.

To measure HMGB1 release, a Lumit™ HMGB1 immunoassay kit (Promega) was used. CT1BA5 cells were trypsinized and seeded in a 96-well plate at a density of 20,000 cells per well. The cells were incubated at 37 °C for 24 h. Following incubation, the media in each well was replaced with fresh media containing IC_{25}, IC_{50}, and IC_{75}equivalent concentrations of the respective V-LNPs. After an additional 24 h incubation, the cells were

irradiated with 690 nm light from the top of the plate at a fluence of 20 J/cm² and an irradiance of 18 mW/cm². 20 µL of 5 X HMGB1 antibody solution (containing Anti-hHMGB1 mAV-SmBiT and Anti-hHMGB1 mAB-LgBiT) was then added to each well and incubated for 60 - 90 min. Following antibody incubation, 25 µL of a 1:20 dilution of Lumit™ detection substrate was added to each well, and the plate was mixed using a shaker at 300–500 RPM. The plate was then incubated for 3–5 min, and luminescence readings were recorded using a multi-plate reader.

For the 6620c1 cell line, all ICD markers were quantified as described above using only IC_{50} BPD equivalent concentration of all the V-LNPs.

Dendritic cell maturation: Spleens were harvested from two euthanized C57BL/6 mice under sterile conditions in accordance with IACUC-approved protocols. Spleens were aseptically dissected and placed in cold RPMI 1640 medium (Sigma) supplemented with 2% FBS. The tissues were minced using sterile surgical scissors and enzymatically digested in RPMI containing 1 mg/mL Collagenase D and 0.1 mg/mL DNase I (Sigma-Aldrich) for 20–30 min at 37 °C with gentle shaking. The resulting suspension was passed through a 70 µm cell strainer (Corning) to obtain a single-cell suspension. Red blood cells (RBCs) were lysed using 1X RBC lysis buffer (Fisher Scientific) for 5 min at room temperature. To minimize nonspecific antibody binding, the cell suspension was incubated with 50 µL of TruStain Fc™ (BioLegend, 0.5 mg/mL, diluted 1:5 in PAB on ice in the dark for 15 min. Cells were then stained with primary anti-CD11c antibody (8.62 mg/mL; 1:200 dilution in PAB; Bio X Cell) for 15 minon ice, washed, and centrifuged at 1500 rpm for 5 min. Cells were resuspended and incubated with secondary anti-CD11c antibody (8.54 mg/mL; 1:500 dilution in PAB; Bio X Cell) for 15 min on ice. After another centrifugation step at 1500 rpm for 5 min, cells were resuspended in FACS buffer, and flow cytometry–based cell sorting was performed to isolate $CD11c^+$ dendritic cells.

Separately, CT1BA5 cells were cultured to ~70% confluence, trypsinized, and seeded at a density of 45,000 cells per well in 6-well plates. After 24 h, cells were treated with BPD equivalent IC_{50} concentrations of LNP BPD-Cholesterol. Following an additional 24h incubation, cells were irradiated using a 690 nm light at a fluence of 20 J/cm² and irradiance of 18 mW/cm². Treated CT1BA5 cells were then incubated for another 24 h before co-culture.Isolated $CD11c^+$ dendritic cells were seeded at 45,000 cells per well and co-cultured with the pre-treated CT1BA5 cells. As controls, one group of dendritic cells was cultured alone, while another was treated with 200 ng/mL lipopolysaccharide (LPS; Invitrogen) to induce maturation. After 24 h of co-culture, cells (both tumor and dendritic cells) were harvested, centrifuged, and resuspended in TruStain (1:5 dilution in PAB) to block Fc receptors, followed by incubation on ice for 10 min. Cells were washed with PAB, centrifuged, and stained with a cocktail of primary antibodies against CD11c, CD80, and CD86 (1:200 dilution in PAB; Bio X Cell) for 15 min on ice. Following primary antibody staining, cells were washed, centrifuged, and resuspended in a secondary antibody mixture targeting CD11c, CD80, and CD86 (1:500 dilution in PAB) for 15 min on ice. Finally, cells were centrifuged and resuspended in FACS buffer. Flow cytometry analysis was performed to assess dendritic cell maturation based on CD80 and CD86 expression within the $CD11c^+$ population.

Tumor homogenization and flow cytometry: CT1BA5 tumors were processed into single-cell suspensions using enzymatic digestion followed by density gradient separation. One day prior to the experiment, digestion buffer (4 mL per tumor) was prepared in RPMI 1640 (Corning) supplemented with 10 mM HEPES (Cytiva), 2% FBS, 1 mM $MgCl_2$, and 1 mM $CaCl_2$. Percoll gradients were prepared by mixing Percoll (Cytiva) with 10× PBS

(Corning) at a 9:1 ratio to generate Percoll-PBS. A 44% (top) Percoll layer was made by mixing Percoll-PBS with RPMI (4.4:5.6), and a 67% (bottom) Percoll layer was prepared by mixing Percoll-PBS with 1× PBS (6.7:3.3). Stop medium consisted of RPMI with 2 mM EDTA and 2% calf serum. On the day of the experiment, complete digestion buffer was prepared by adding Liberase TL (Sigma; final concentration 50 µg/mL) and DNase I (Sigma; final concentration 0.1 mg/mL) to the digestion buffer. CT1BA5 tumors were minced on ice and transferred to 50 mL conical tubes containing 4 mL complete digestion buffer, followed by incubation at 37 °C for 40 min at 180 rpm. Digested tissue was passed through a cell strainer using the plunger of a 3 mL syringe, and the strainer was rinsed with stop medium to terminate enzymatic activity. Cell suspensions were centrifuged at 2000 rpm for 5 min at 4 °C, resuspended in 5 mL 44% Percoll, and layered over 5 mL 67% Percoll in a 15 mL tube. Gradients were centrifuged at room temperature (2000 rpm, 20 min, acceleration 9, no brake). The leukocyte layer at the interface was collected (≤3 mL), diluted to 14 mL with stop medium, and centrifuged again (2000 rpm, 10 min, 4 °C). Cell pellets were resuspended in 500 µL RPMI with 2% calf serum, and viable cells were counted prior to flow cytometry analysis. Single-cell suspensions from tumor tissue were plated in 96-well plates. Plates were centrifuged (2000 rpm, 1 min, 4 °C), supernatants discarded, and cells were resuspended in 100 µL MACS buffer and combined with the corresponding cells. Samples were incubated for 10 min on ice. Surface staining cocktails (500 µL total, prepared according to the flow cytometry panel) were prepared in advance. Cells were centrifuged (2000 rpm, 1 min, 4 °C), washed twice with 200 µL FACS buffer, and resuspended in 50 µL staining cocktail per sample. Staining was performed for 20 min on ice, followed by 2.5 washes with FACS buffer. Cells were fixed in 100 µL of 1% paraformaldehyde (PFA) for 10 min at room temperature, centrifuged (2500 rpm, 1 min, 4 °C), washed once with 200 µL FACS buffer, and resuspended in 200 µL FACS buffer. Samples were filtered into flow cytometry tubes, and 100 µL of each sample was acquired on a flow cytometer.

Principal Component Analysis (PCA): PCA was performed to reduce thedimensionality of allthe data generated and to identify relationships between thedifferent V-LNP attributes (type and quantity of ROS generation, subcellular localization, phototoxicity), and their ability to induce exposure of ICD markers. The analysis was conducted using GraphPad Prism v10.4.1. To determine the contribution of each principal component (PC) to the total variance, eigenvalues were computed. PCA scores were calculated to position each formulation within the PCA space, enabling clustering analysis based on shared characteristics. A loadings plot was used to assess the contribution of individual variables to each PC. Additionally, a Pearson correlation matrix was generatedto individually evaluate associations between variables and a heatmap was generated to visualize these correlations.

Supporting Data:

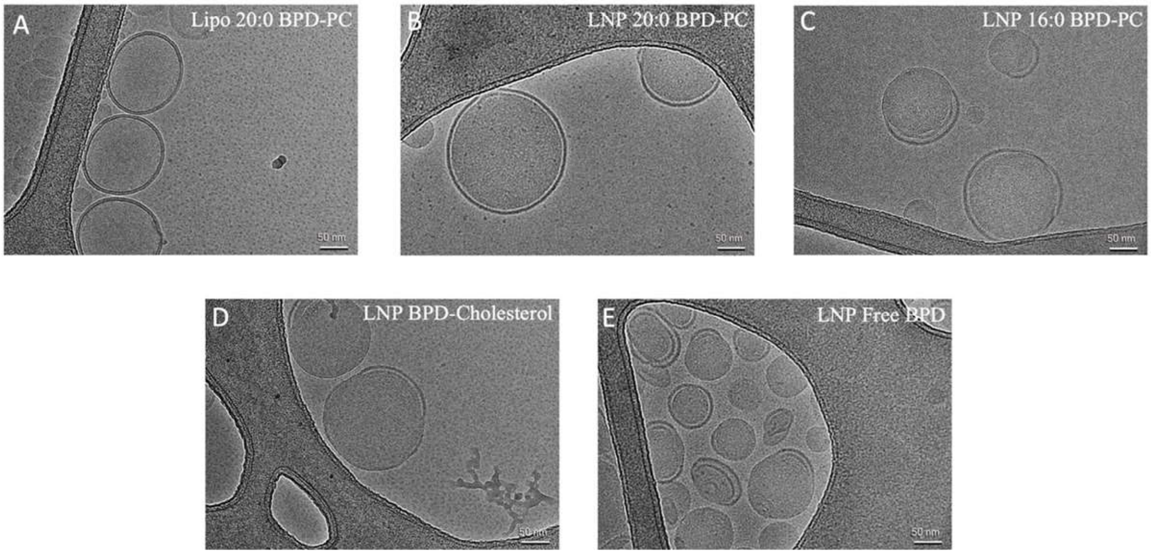

Figure S1: Representative cryo-Transmission Electron Microscopy (TEM) images of V-LNPs. (A) Lipo 20:0 BPD-PC, (B) LNP 20:0 BPD-PC, (C) LNP 16:0 BPD-PC, (D) LNP BPD-Cholesterol, and (E) LNP Free BPD. Scale bar, 50nm.

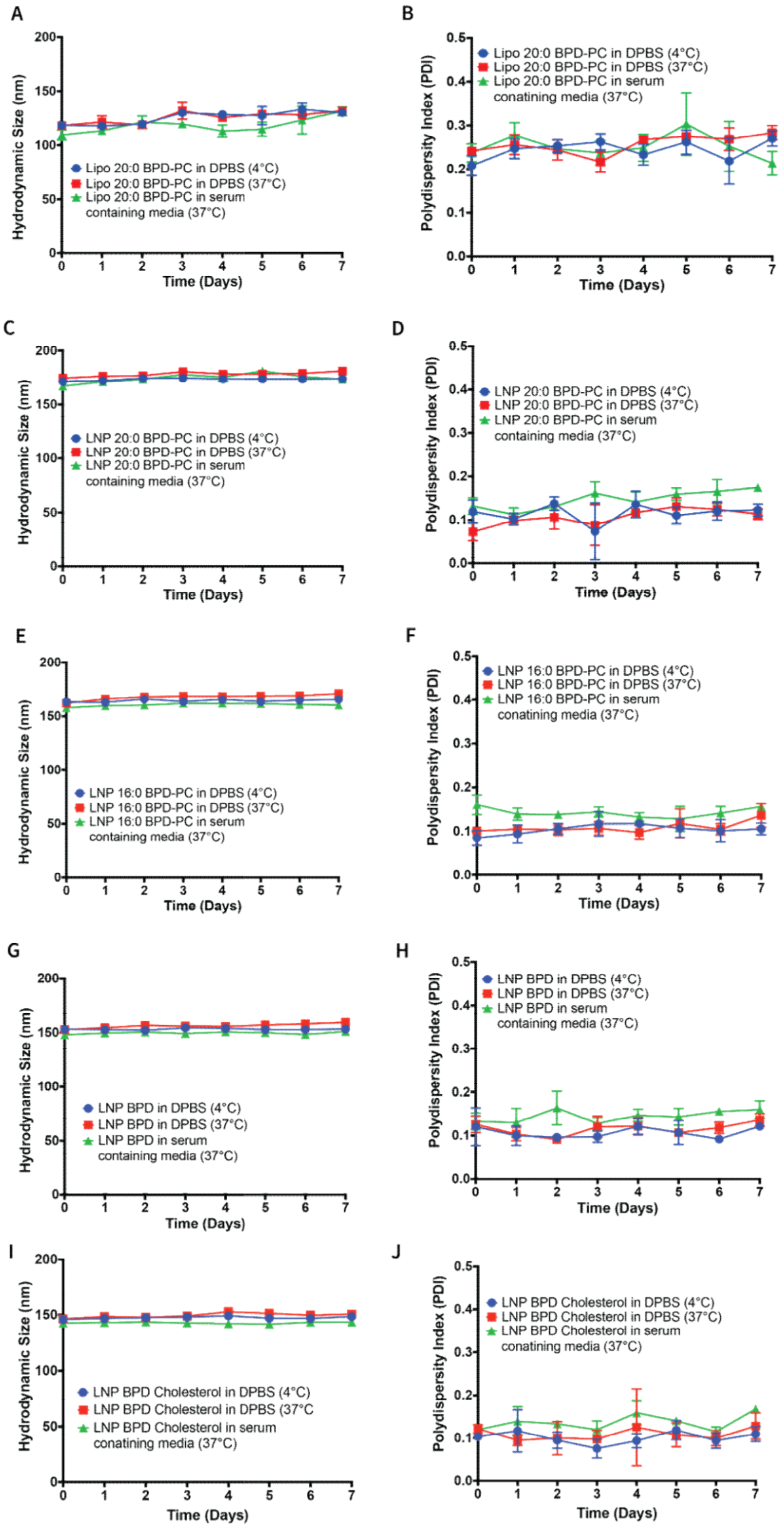

Figure S2: Physical stability study of all the formulations were preformed over the course of 7 days at 37°C in DPBS and serum and 4°C in DPBS. (A and B) shows the hydrodynamic size and polydispersity index of Lipo20:0 BPD-PC. (C and D) shows the hydrodynamic size and polydispersity index of LNP 20:0 BPD-PC and (E and F) shows the hydrodynamic size and polydispersity index of LNP 16:0 BPD-PC.(G and H) shows the hydrodynamic size and polydispersity index of LNP Free BPD.(I and J) shows the hydrodynamic size and polydispersity index of LNP BPD-Cholesterol. Data for LNP 20:0 BPD-PC and Lipo 20:0 BPD-PC was adated from our previous study.[6]

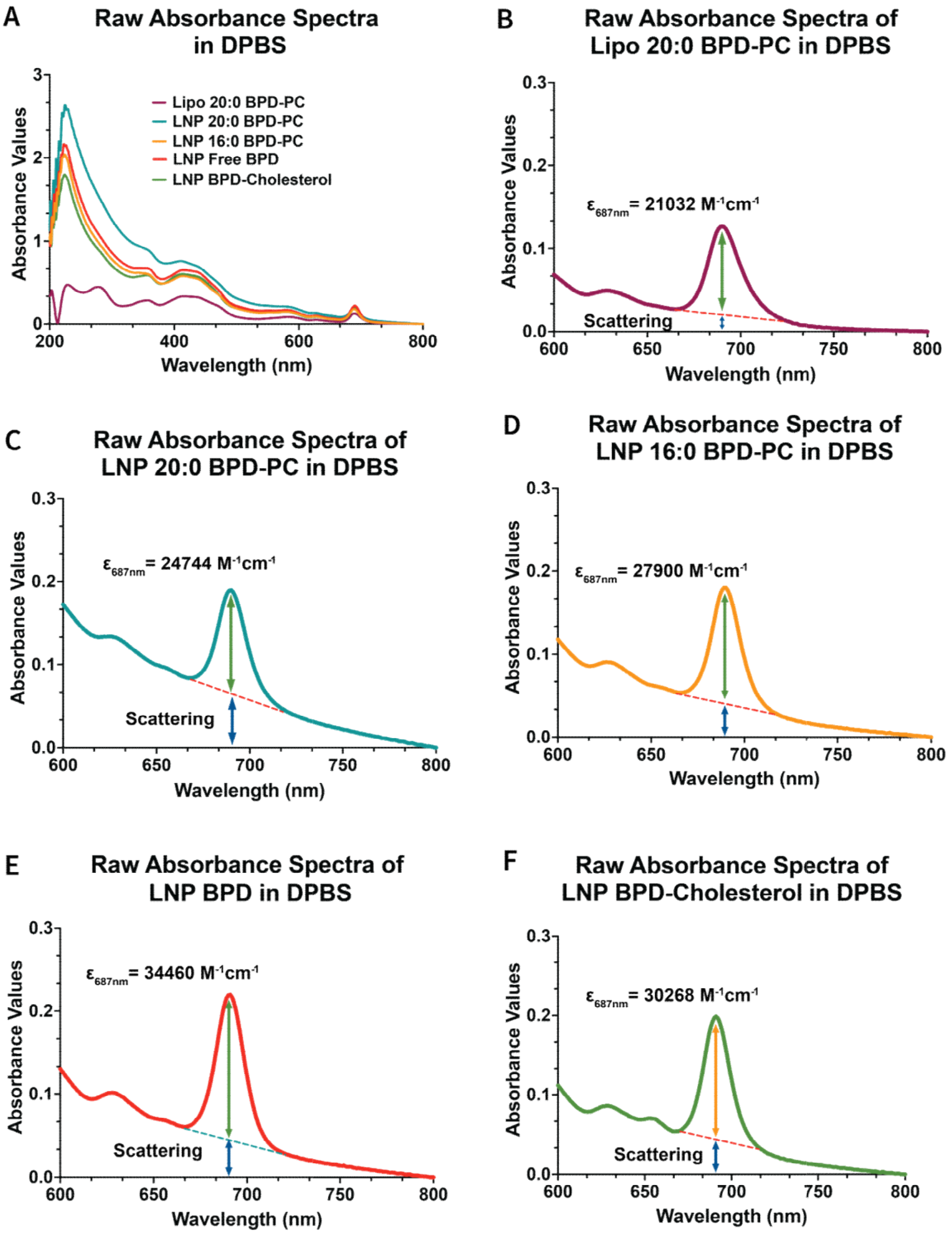

Figure S3: (A) Raw absorbance spectra of 5 μM BPD equivalent concentrationof different V-LNPs in DPBS. Raw absorbance spectra (zoomed) of 5 μM BPD equivalent of the (B) Lipo 20:0 BPD-PC, (C)LNP 20:0 BPD-PC, (D)LNP 16:0 BPD-PC, (E) LNPBPD,and (F) LNP BPD-Cholesterol in DPBS for calculating ΔAbs_{687} nm and ε_{687} nm.Spectrafor LNP 20:0 BPD-PC and Lipo 20:0 BPD-PC was adapted from our previously published article.[6]

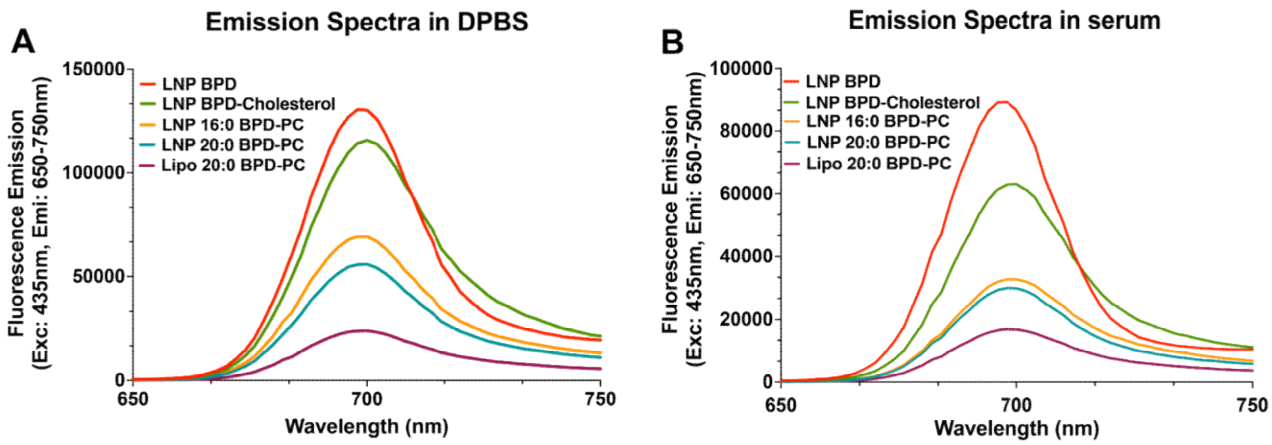

Figure S4: Fluorescence emission spectra of 5 µM BPD equivalent concentration of LNP 20:0 BPD-PC, LNP 16:0 BPD-PC, LNP BPD-Cholesterol, LNPBPD, and Lipo 20:0 BPD-PC in (A) DPBS and (B) serum. Data for LNP 20:0 BPD-PC and Lipo 20:0 BPD-PC was adapted from our previously published article.[6]

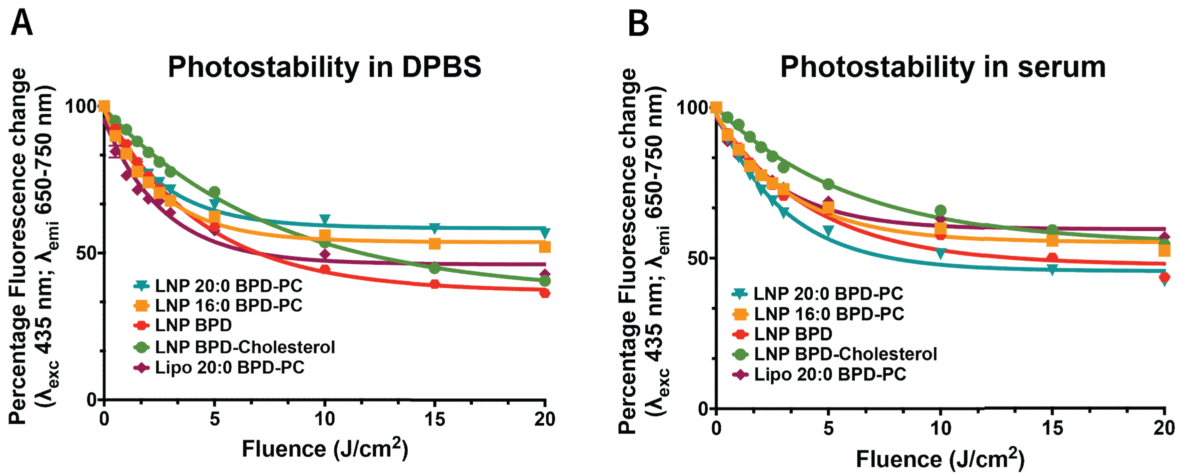

Figure S5: Relative changes in the peak fluorescence emission value upon photoirradiation in (A) DPBS and (B) serum. Photoirradiation was performed using 690 nm wavelength of LED light with fluences of 0 J/cm², 0.5 J/cm², 1 J/cm², 1.5 J/cm², 2 J/cm², 2.5 J/cm², 3 J/cm², 5 J/cm², 10 J/cm², 15 J/cm² and 20 J/cm² at 18 mW/cm^2 irradiance. Data for LNP 20:0 BPD-PC and Lipo 20:0 BPD-PC was adapted from our previously published article.[6]

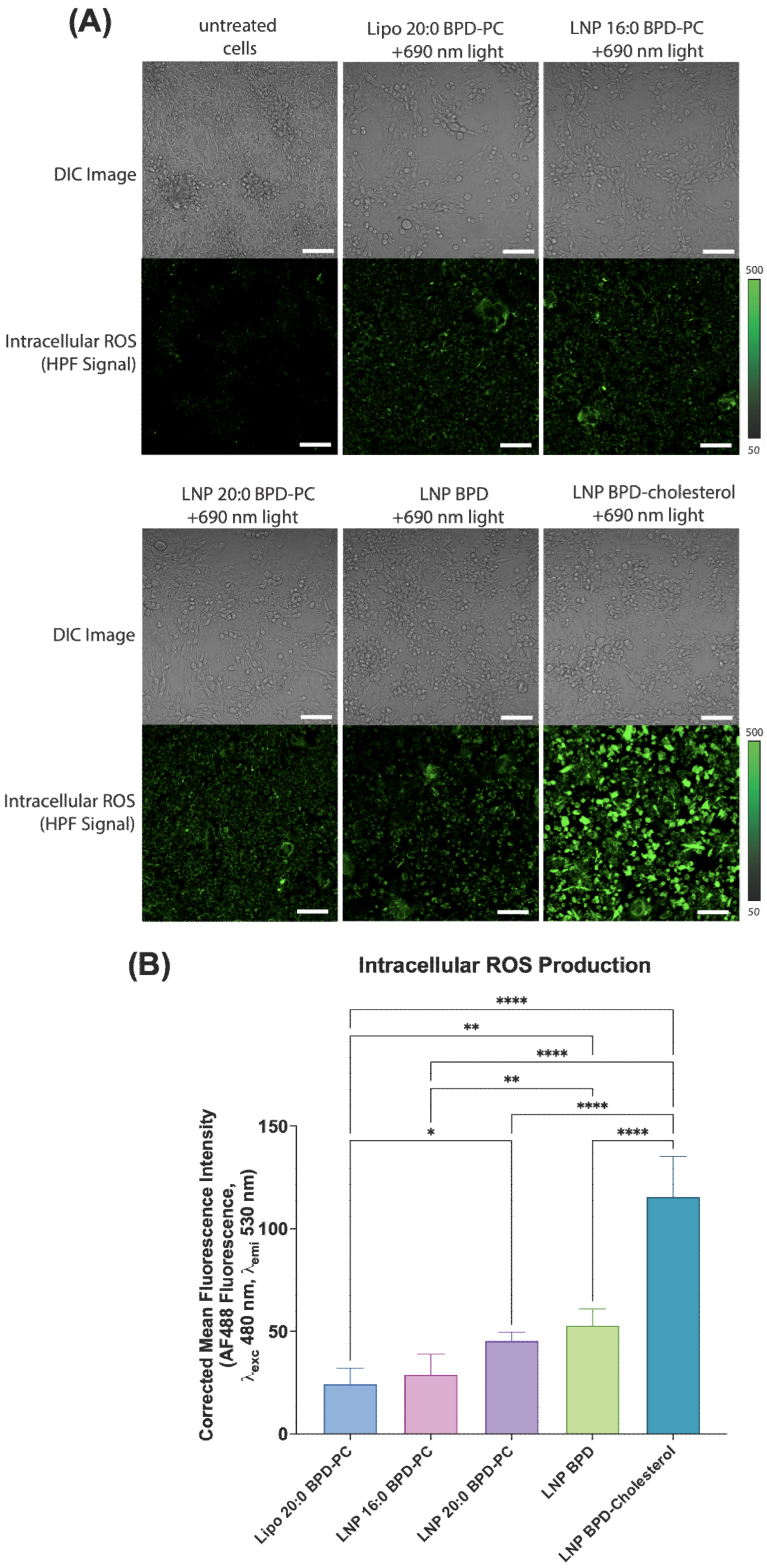

Figure S6: Intracellular production of Type I ROS in CT1BA5 cells treated with V-LNP formulations. (A) Representative confocal fluorescence images showing Type I ROS generation detected by HPF following treatment with the indicated V-LNPs and light irradiation. (B) Quantification of corrected mean fluorescence intensity for Type I ROS production corresponding to each formulation (Quantified using ImageJ, all data was are presented as mean ± S.D., (n=6). Statistical significance was calculated using a one-way ANOVA test on GraphPad Prism v10.4.1, *: $P < 0.1$, **: $P < 0.01$, ***: $P < 0.001$, ****: $P < 0.0001$)

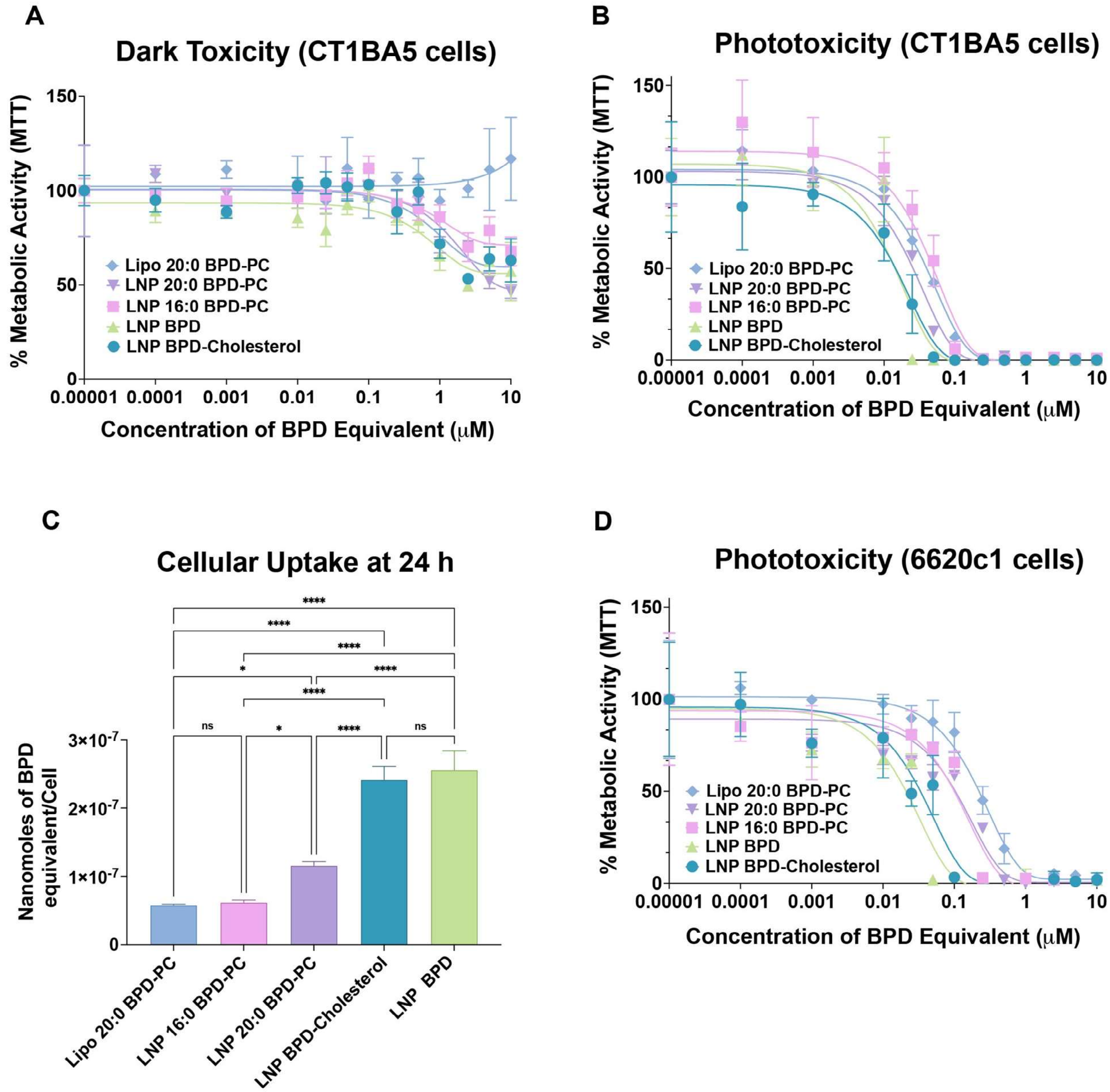

Figure S7: Metabolic activity of CT1BA5 cells as determined by the MTT assay following (A) no PDT and (B) PDT using V-LNPs activated by 690 nm light with a fluence of 20 J/cm^2. (C) Comparison of CT1BA5 cellular uptake of V-LNPs at 24 h. Dark toxicity and phototoxicity data for LNP 20:0 BPD-PC and Lipo 20:0 BPD-PC was adapted from our previously published article.[6](D) Metabolic activity of 6620c1 cells as determined by MTT assay following PDT usingV-LNPs activated by 690 nm light with a fluence of 20 J/cm^2. (All data are presented as mean ± S.D., (n=6). Statistical significance was calculated using a one-way ANOVA test on GraphPad Prism v10.4.1, *: $P < 0.1$, **: $P < 0.01$, ***: $P < 0.001$, ****: $P < 0.0001$)

Immunogenic Cell Death Analysis: Translocation/Release of Calreticulin in CT1BA5 cells

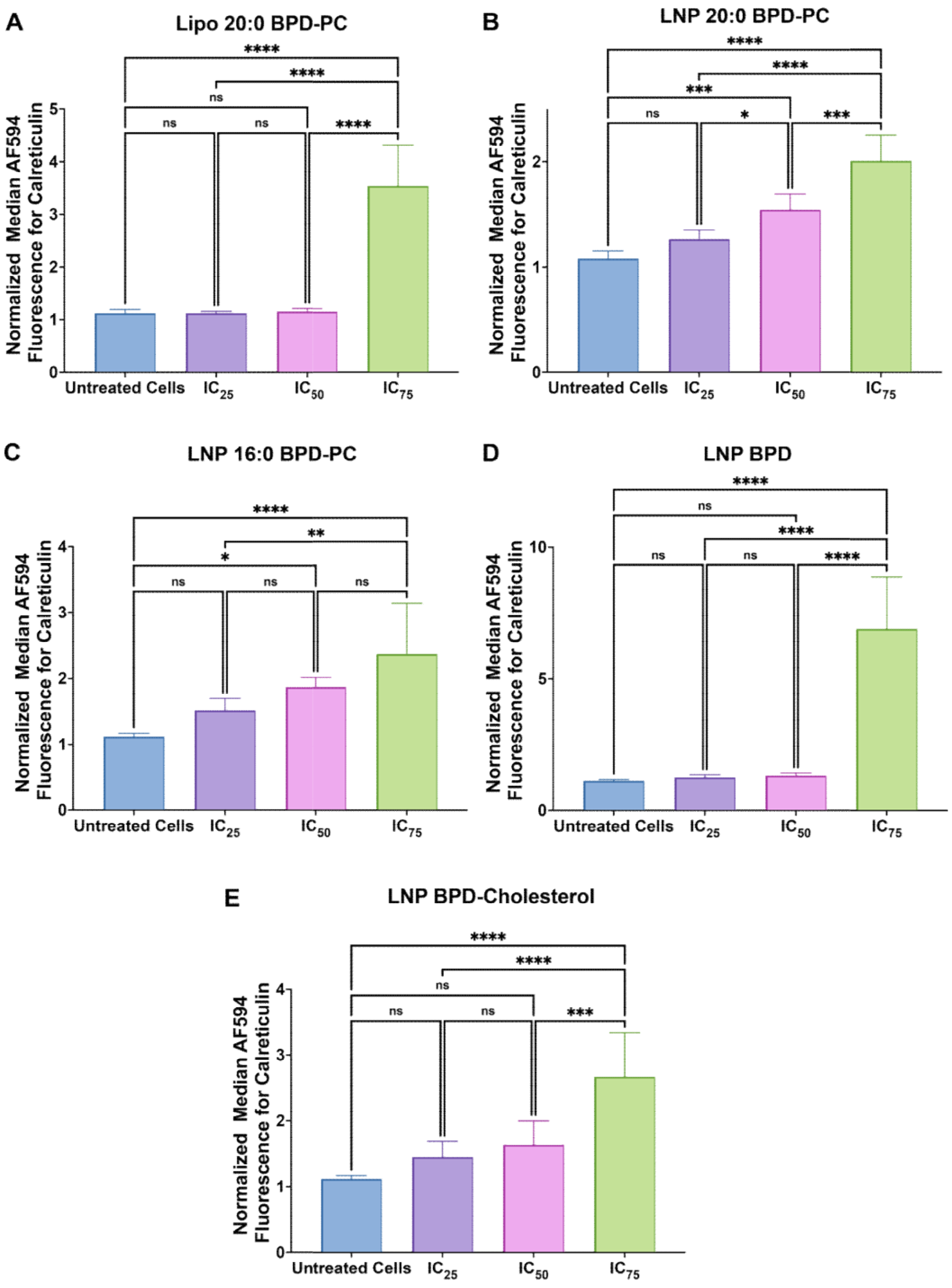

Figure S8: Exposure of the immunogenic cell death marker calreticulin in CT1BA5 cells post PDT using 690 nm light with a fluence of 20 J/cm^2 and IC_{25}, IC_{50} and IC_{75} concentrations of (A) Lipo 20:0 BPD-PC, (B) LNP 20:0 BPD-PC, (C) LNP 16:0 BPD-PC, (D) LNP BPD, and (E) LNP BPD-Cholesterol. IC_{25} and IC_{50} data for LNP 20:0 BPD-PC and Lipo 20:0 BPD-PC was adapted from our previously published article.[6](All data was normalized to untreated cells and are presented as mean ± S.D., (n=6). Statistical significance was calculated using a one-way ANOVA test on GraphPad Prism v10.4.1, *: $P < 0.1$, **: $P < 0.01$, ***: $P < 0.001$, ****: $P < 0.0001$)

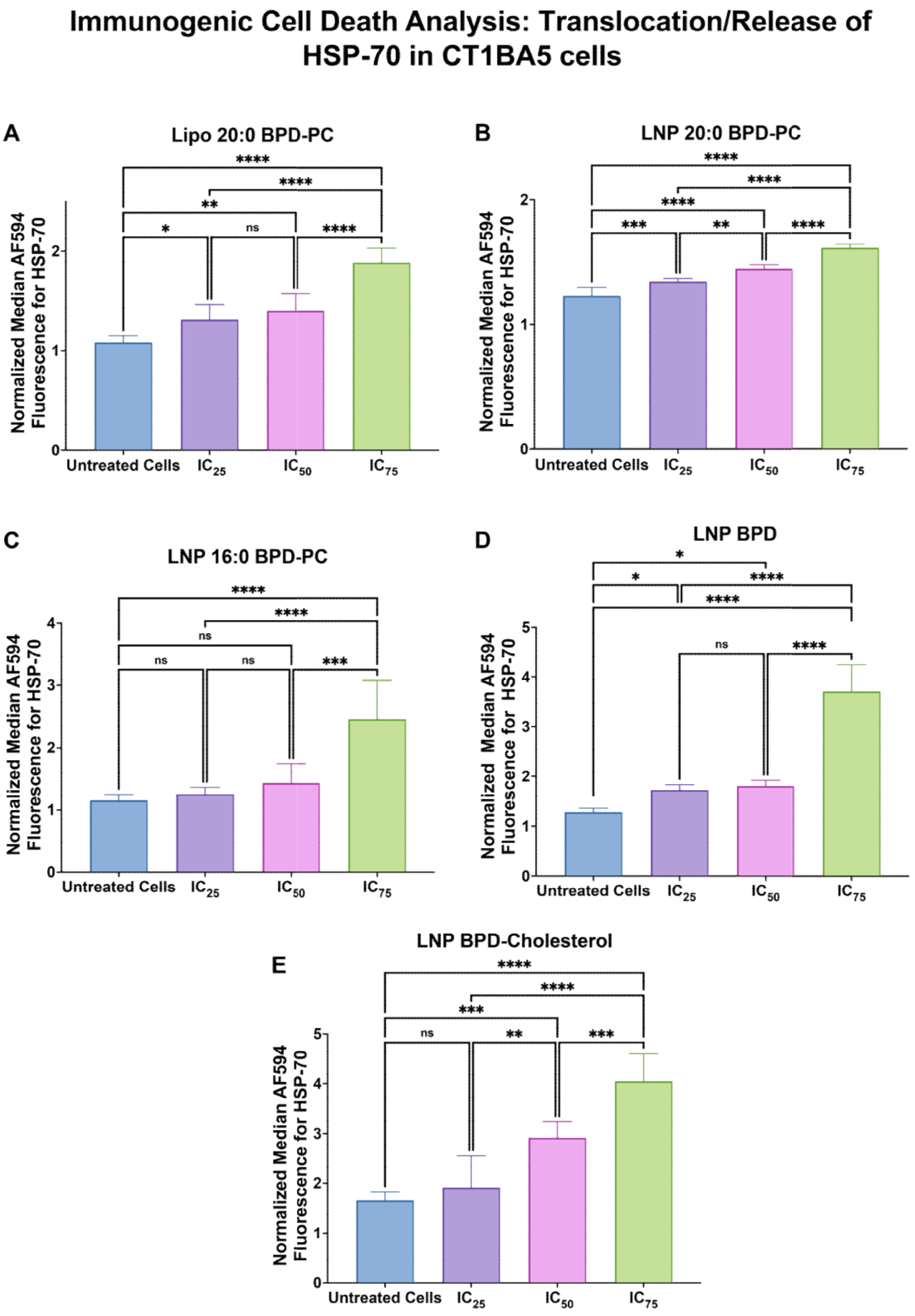

Figure S9: Exposure of the immunogenic cell death marker HSP-70 in CT1BA5 cells post PDT using 690 nm light with a fluence of 20 J/cm^2 and IC_{25}, IC_{50} and IC_{75} concentrations of (A) Lipo 20:0 BPD-PC, (B) LNP 20:0 BPD-PC, (C) LNP 16:0 BPD-PC, (D) LNP BPD, and (E) LNP BPD-Cholesterol. (All data was normalized to untreated cells and are presented as mean ± S.D., (n=6). Statistical significance was calculated using a one-way ANOVA test on GraphPad Prism v10.4.1, *: $P < 0.1$, **: $P < 0.01$, ***: $P < 0.001$, ****: $P < 0.0001$)

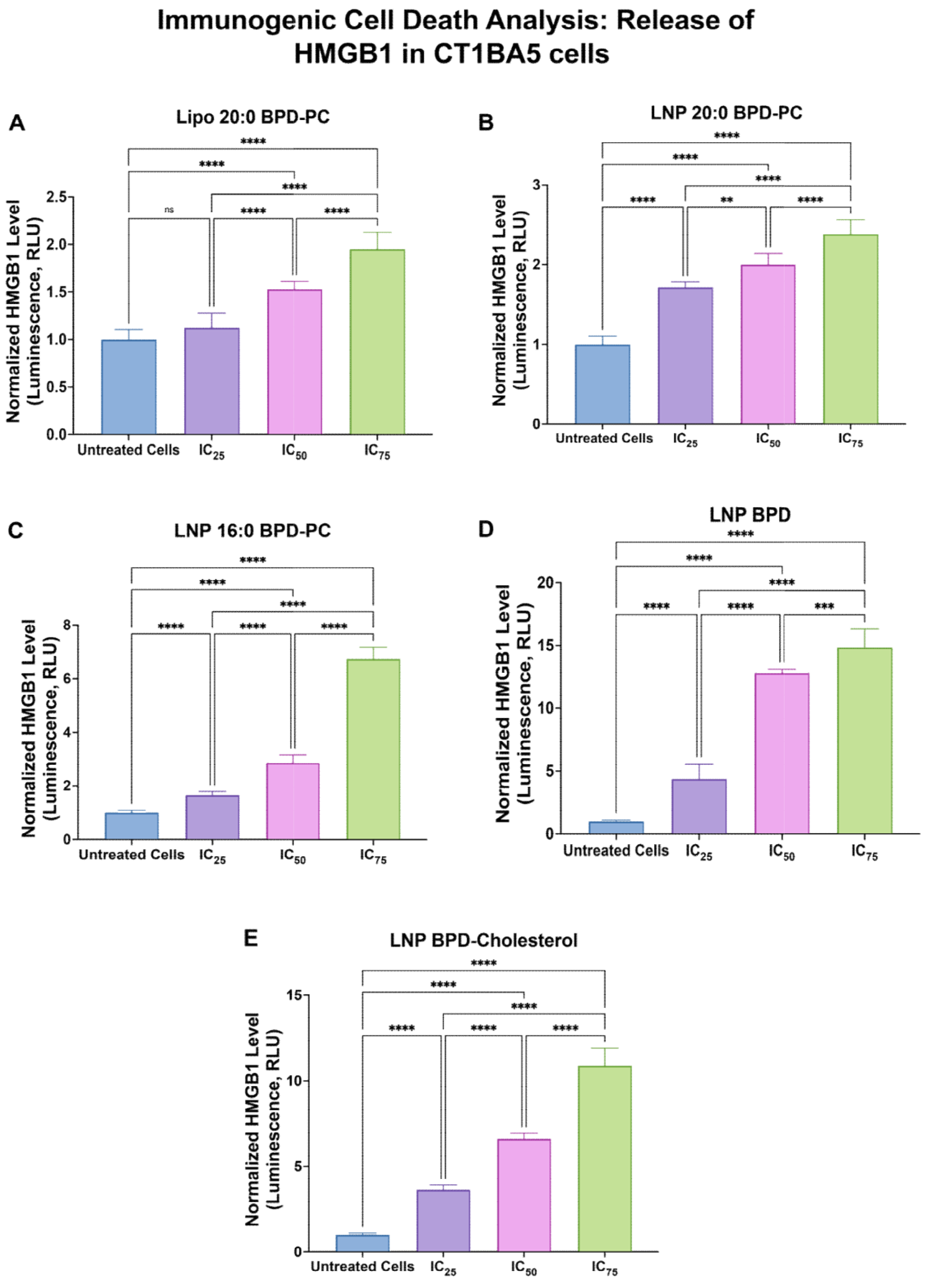

Figure S10: Exposure of the immunogenic cell death marker HMGB1 in CT1BA5 cells post PDT using 690 nm light with a fluence of 20 J/cm^2 and IC_{25}, IC_{50} and IC_{75} concentrations of (A) Lipo 20:0 BPD-PC, (B) LNP 20:0 BPD-PC, (C) LNP 16:0 BPD-PC, (D) LNP BPD, and (E) LNP BPD-Cholesterol. (All data was normalized to untreated cells and are presented as mean ± S.D., (n=6). Statistical significance was calculated using a one-way ANOVA test on GraphPad Prism v10.4.1, *: $P < 0.1$, **: $P < 0.01$, ***: $P < 0.001$, ****: $P < 0.0001$)

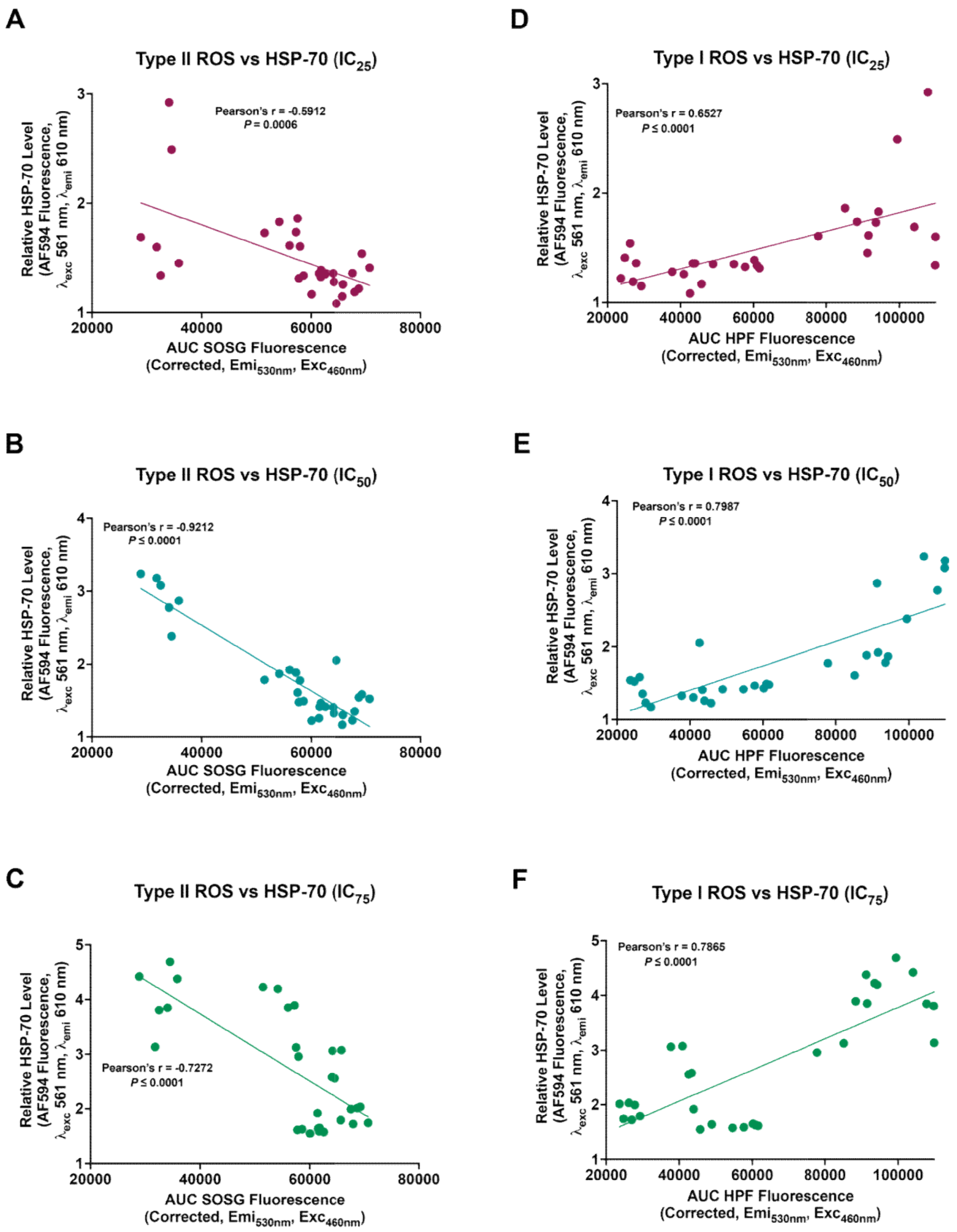

Figure S11: Representative scatter plots showing the relationships between the exposure of the immunogenic cell death marker HSP-70 following PDT of CT1BA5 cells using IC_{25}, IC_{50} and IC_{75} BPD equivalent concentrations of V-LNPs and Type II ROS (A,B,C) orType I ROS (D,E,F). HSP-70 exposure was measured using flow cytometry and is presented as levels relative to untreated cells. Type II ROS is represented by the AUC of the emission curves of the SOSG probe and Type I ROS is represented by the AUC of the emission curves of the HPF probe following 690 nm activation of V-LNPs.

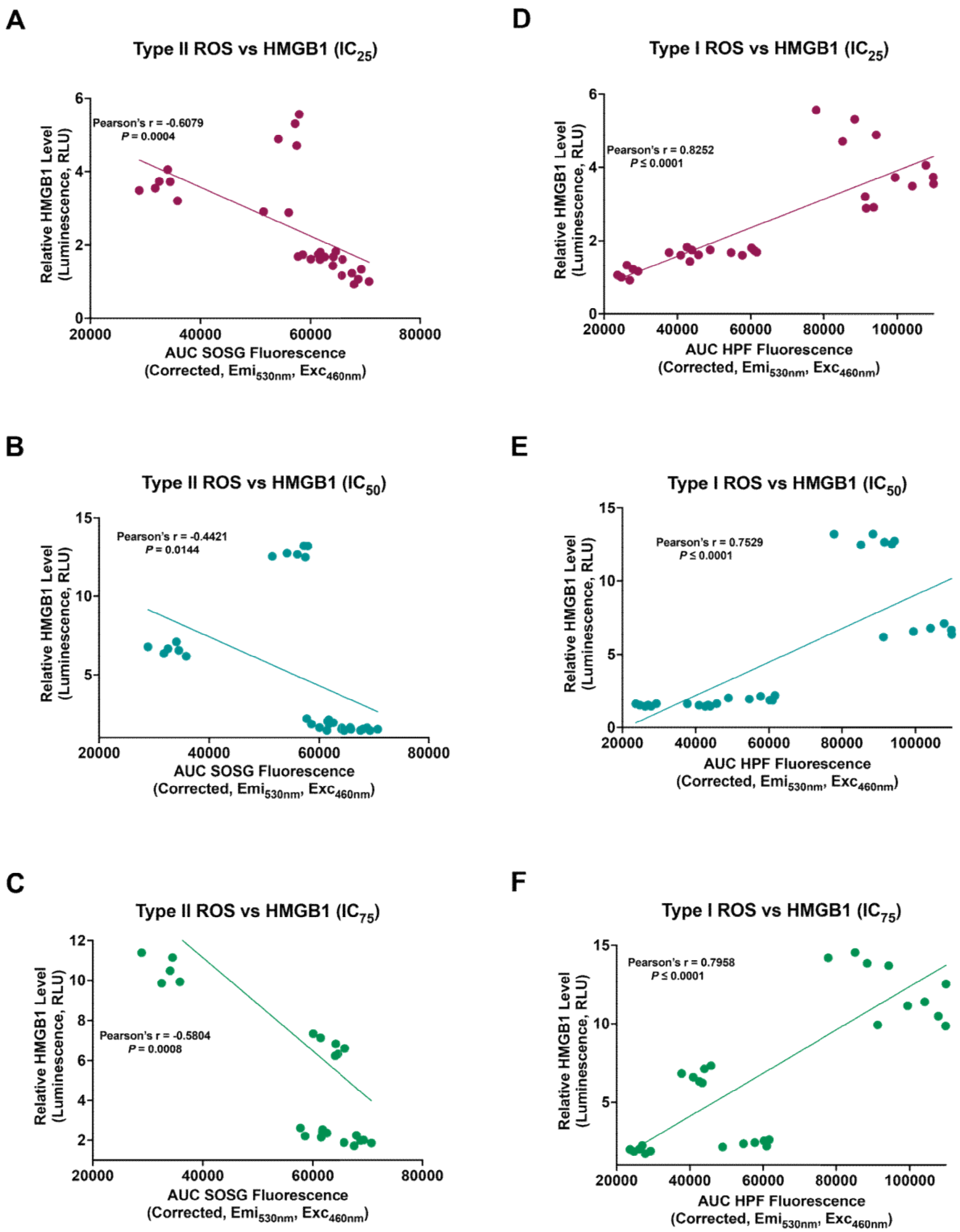

Figure S12: Representative scatter plots showing the relationships between the exposure of the immunogenic cell death marker HMGB1following PDT of CT1BA5 cells using IC_{25}, IC_{50} and IC_{75} BPD equivalent concentrations of V-LNPs and Type II ROS (A,B,C) or Type I ROS (D,E,F). HMGB1 exposure was measured using a Lumit™ bioluminescence kit (Promega)and is presented as levels relative to untreated cells. Type II ROS is represented by the AUC of the emission curves of the SOSG probe and Type I ROS is represented by the AUC of the emission curves of the HPF probe following 690 nm activation of V-LNPs.

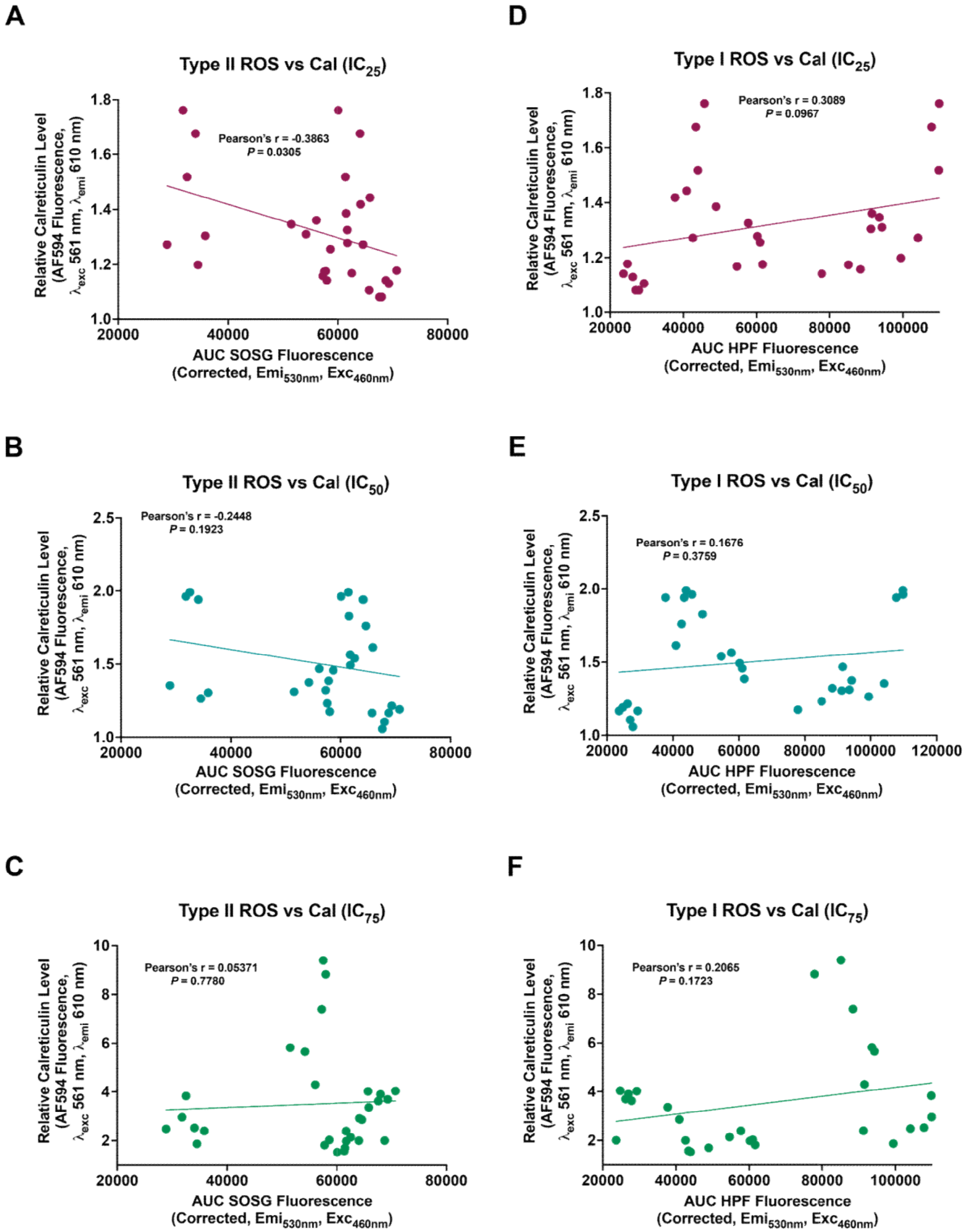

Figure S13: Representative scatter plots showing the relationships between the exposure of the immunogenic cell death marker calreticulinfollowing PDT of CT1BA5 cells using IC_{25}, IC_{50} and IC_{75} BPD equivalent concentrations of V-LNPs and Type II ROS (A,B,C) or Type I ROS (D,E,F).Calreticulin exposure was measured using flow cytometryand is presented as levels relative to untreated cells. Type II ROS is represented by the AUC of the emission curves of the SOSG probe and Type I ROS is represented by the AUC of the emission curves of the HPF probe following 690 nm activation of V-LNPs.

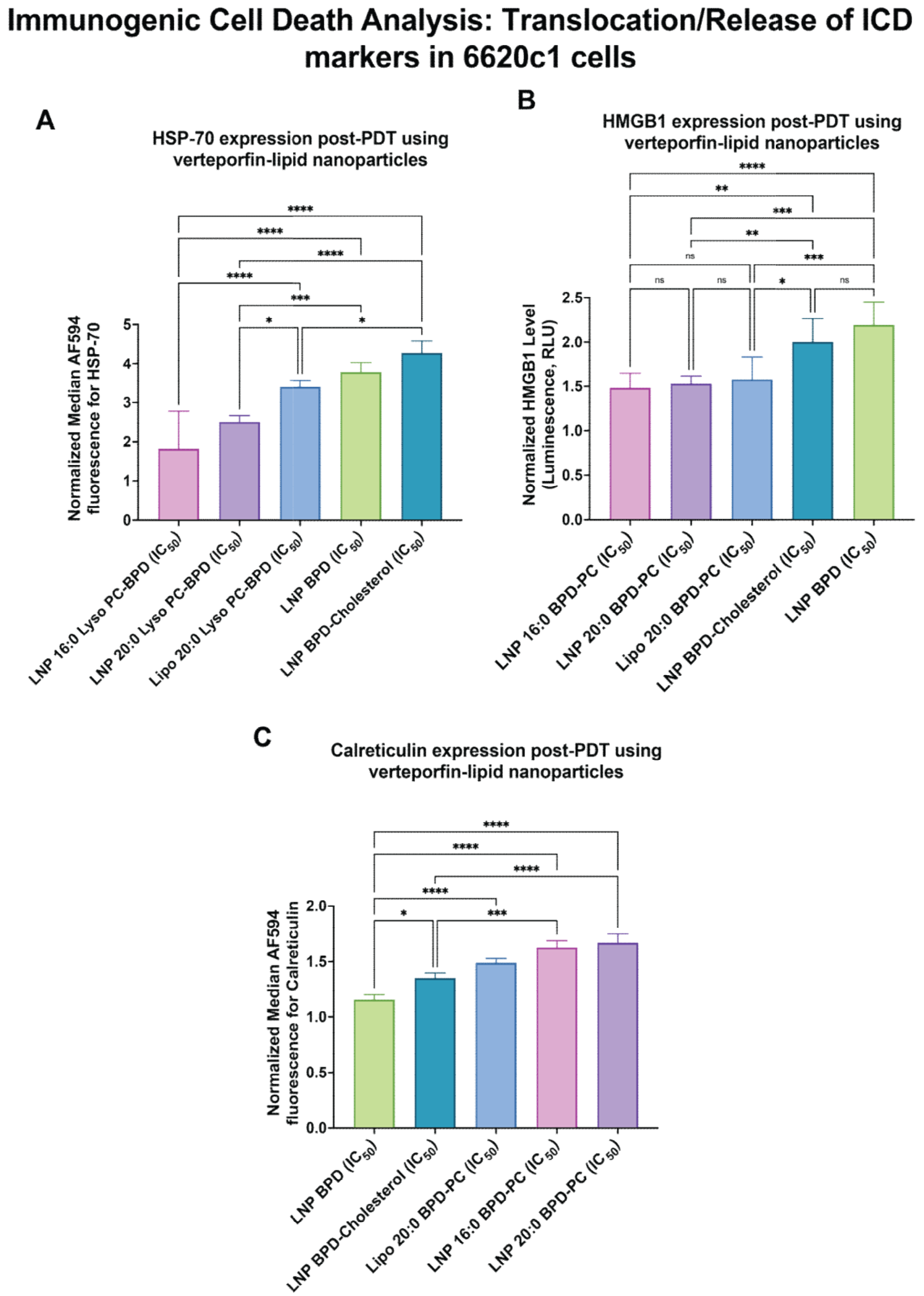

Figure S14: Exposure of the immunogenic cell death markers (A) HSP-70, (B) HMGB1 and (C) calreticulin in 6620c1 cells post-PDT using 690 nm light with a fluence of 20 J/cm^2 and IC_{50} BPD equivalent concentration of V-LNPs. (All data was normalized to untreated cells and are presented as mean ± S.D., (n=6). Statistical significance was calculated using a one-way ANOVA test on GraphPad Prism v10.4.1, *: $P < 0.1$, **: $P < 0.01$, ***: $P < 0.001$, ****: $P < 0.0001$)

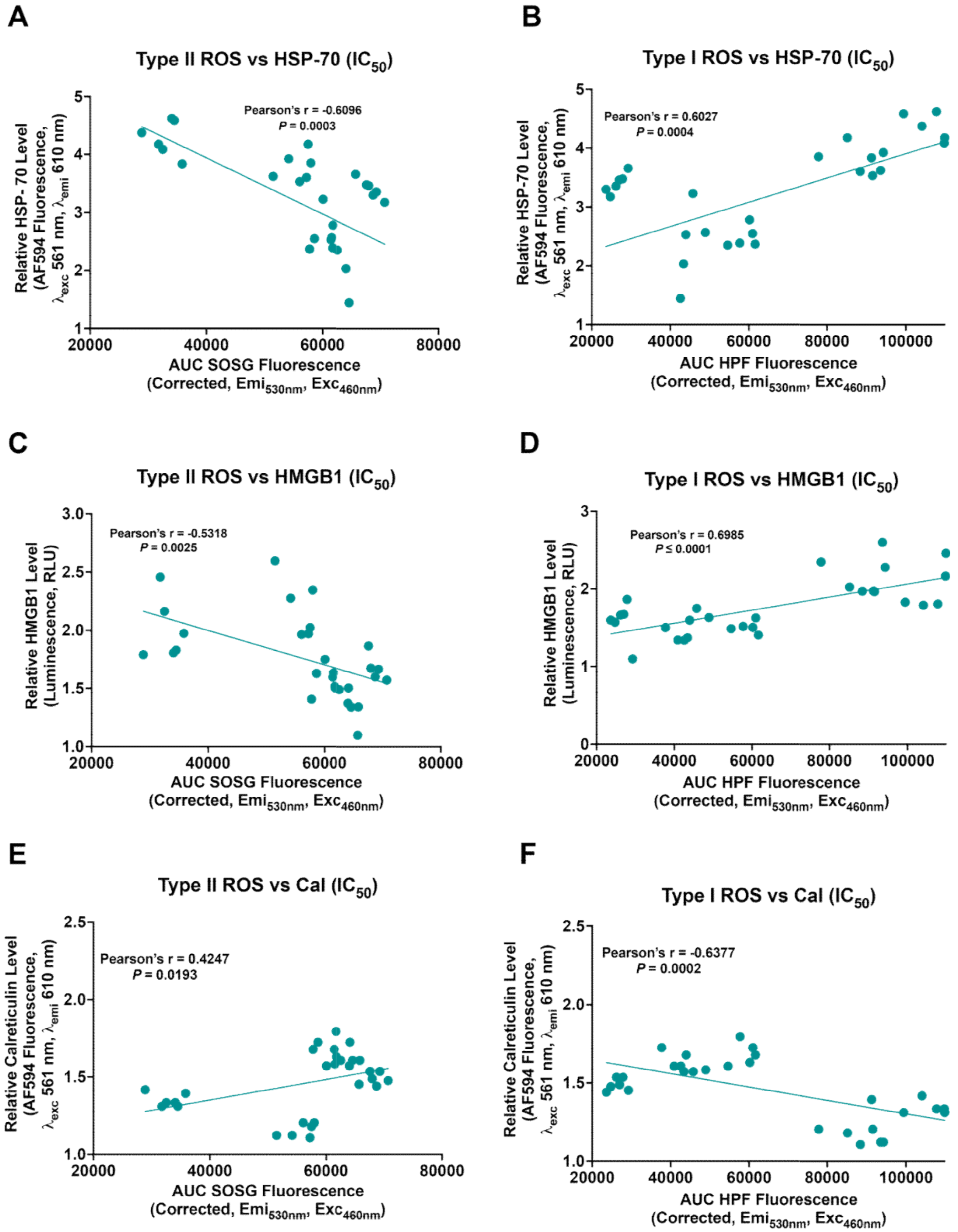

Figure S15: Representative scatter plots showing the relationships between the exposure of the immunogenic cell death marker HSP-70, HMGB-1, and calreticulin (Cal) at IC_{50} BPD equivalent concentrations of V-LNPs in 6620c1 cells and Type II ROS (A,C,E) orType I ROS (B,D,F). HSP-70 and calreticulin were measured using flow cytometry and HMGB1 was measured using a bioluminescence kit (Promega). Type II ROS is represented by the AUC of the emission curves of the SOSG probe and Type I ROS is represented by the AUC of the emission curves of the HPF probe following 690 nm activation of V-LNPs.

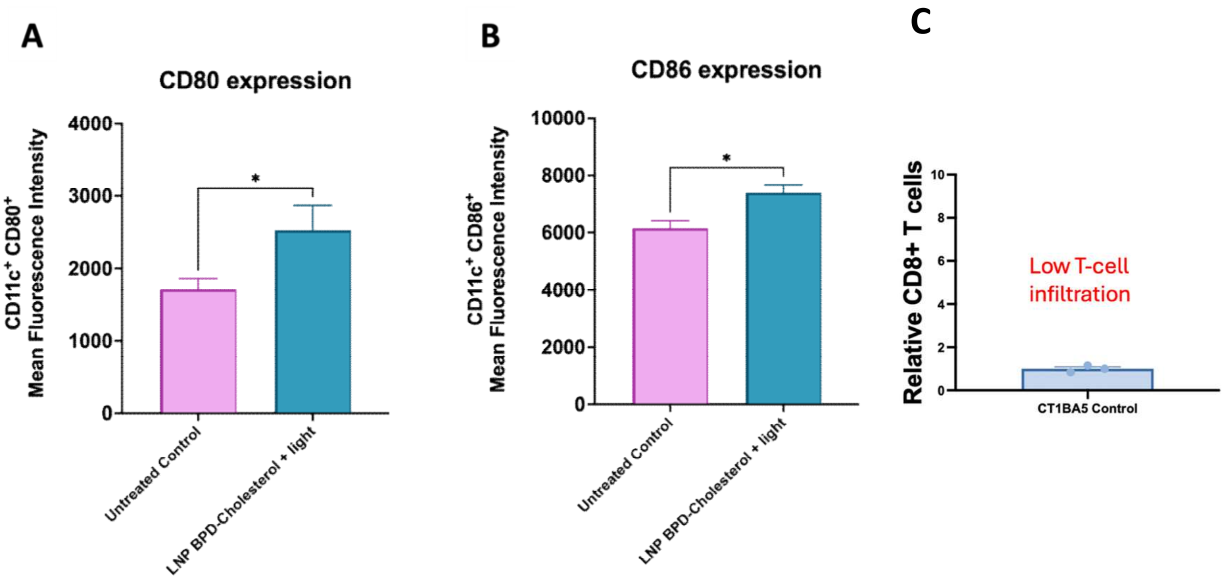

Figure S16: Dendritic cell (DC) activation analysis using expression of co-stimulatory marker (A) CD80 and (B) CD86. DCs were co-cultured with CT1BA5 cells post-PDT (690 nm light, 20 J/cm²) using the IC_{50} BPD-equivalent concentration of LNP BPD-Cholesterol. (All data are presented as mean ± S.D., (n=3). Statistical significance was calculated using a t-test on GraphPad Prism v10.4.1, *: $P < 0.1$). (C) Whole tumor flow cytometry analysis of intratumoral $CD8^+$ T cells in CT1BA5 tumors in C57BL/6mice.

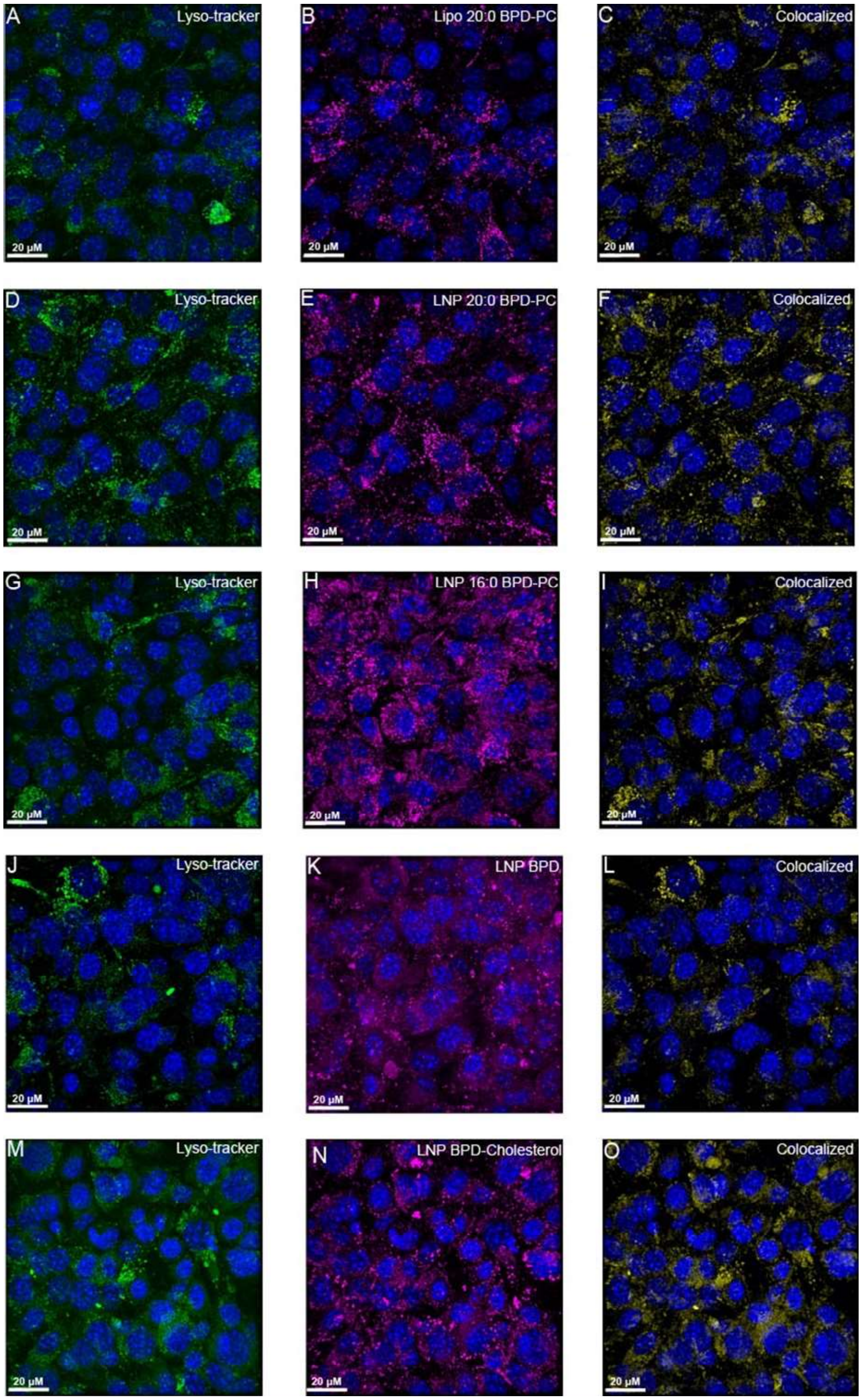

Figure S17: Representative confocal microscopy images of CT1BA5 cells using a 100 × objective after 24 h incubation with Lipo 20:0 BPD-PC (B), LNP 20:0 BPD-PC (E), LNP 16:0 BPD-PC (H), LNPBPD (K), and LNP BPD-Cholesterol (N). Lysosomes were labeled with LysoTracker Green DND-26 (green; A,D,G,J,M). For each formulation, the first two images were merged using Imaris software to identify regions of colocalization (yellow; C, F, I, L, O) between the formulations and lysosomes. The brightness of the images was adjusted for improvedvisualization but all quantitation of co-localization was performed on unmodified images. Scale bars are 20 μm.

WILEY-VCH

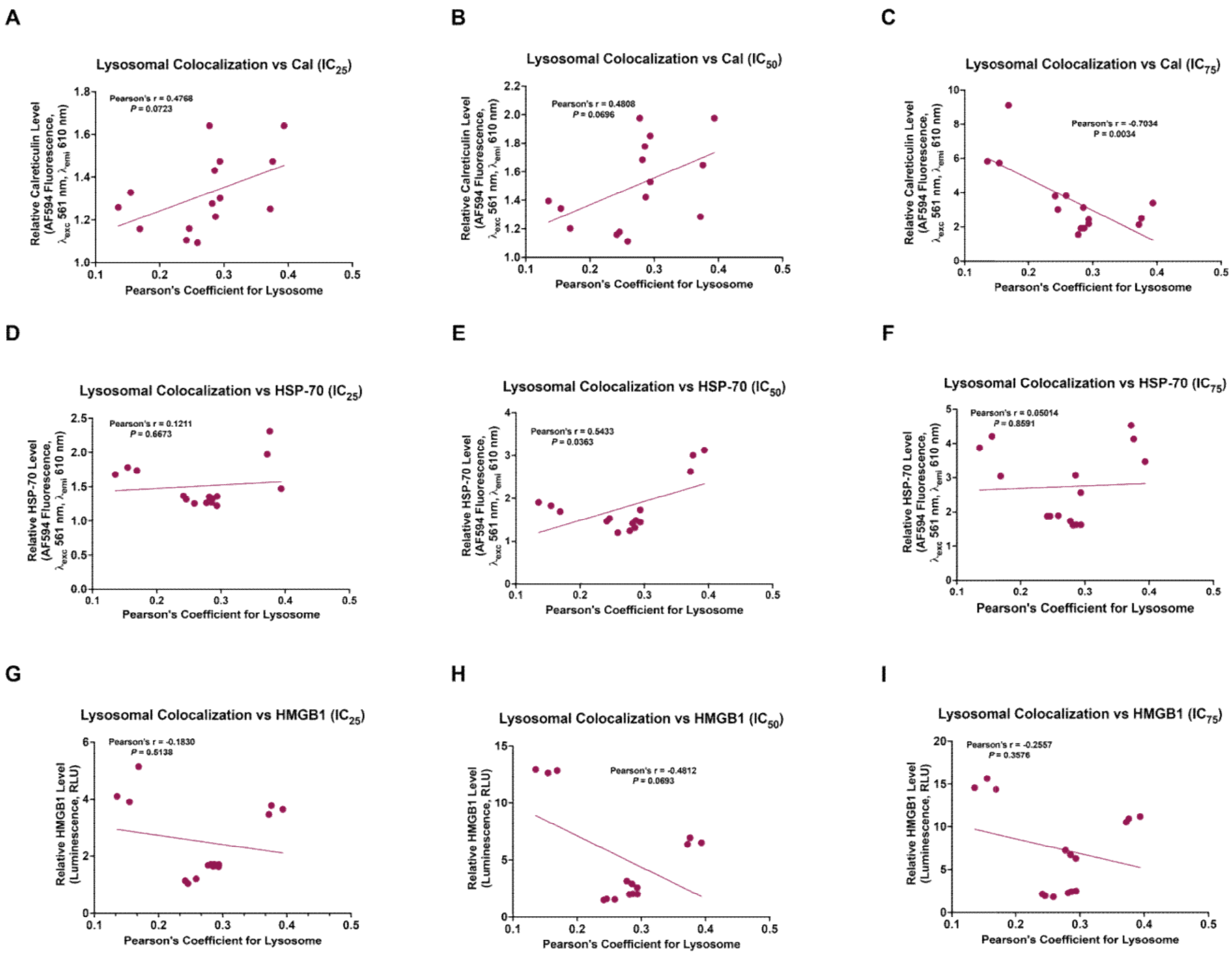

Figure S18: Scatter plots representing the relationships between the exposure of the immunogenic cell death markerscalreticulin (Cal), HSP-70, and HMGB1 in CT1BA5 cells when using IC_{25}, IC_{50} and IC_{75} BPD equivalent concentrations of V-LNPs and the Pearson's coefficient obtained for their co-localizationwith LysoTracker Green DND-26.

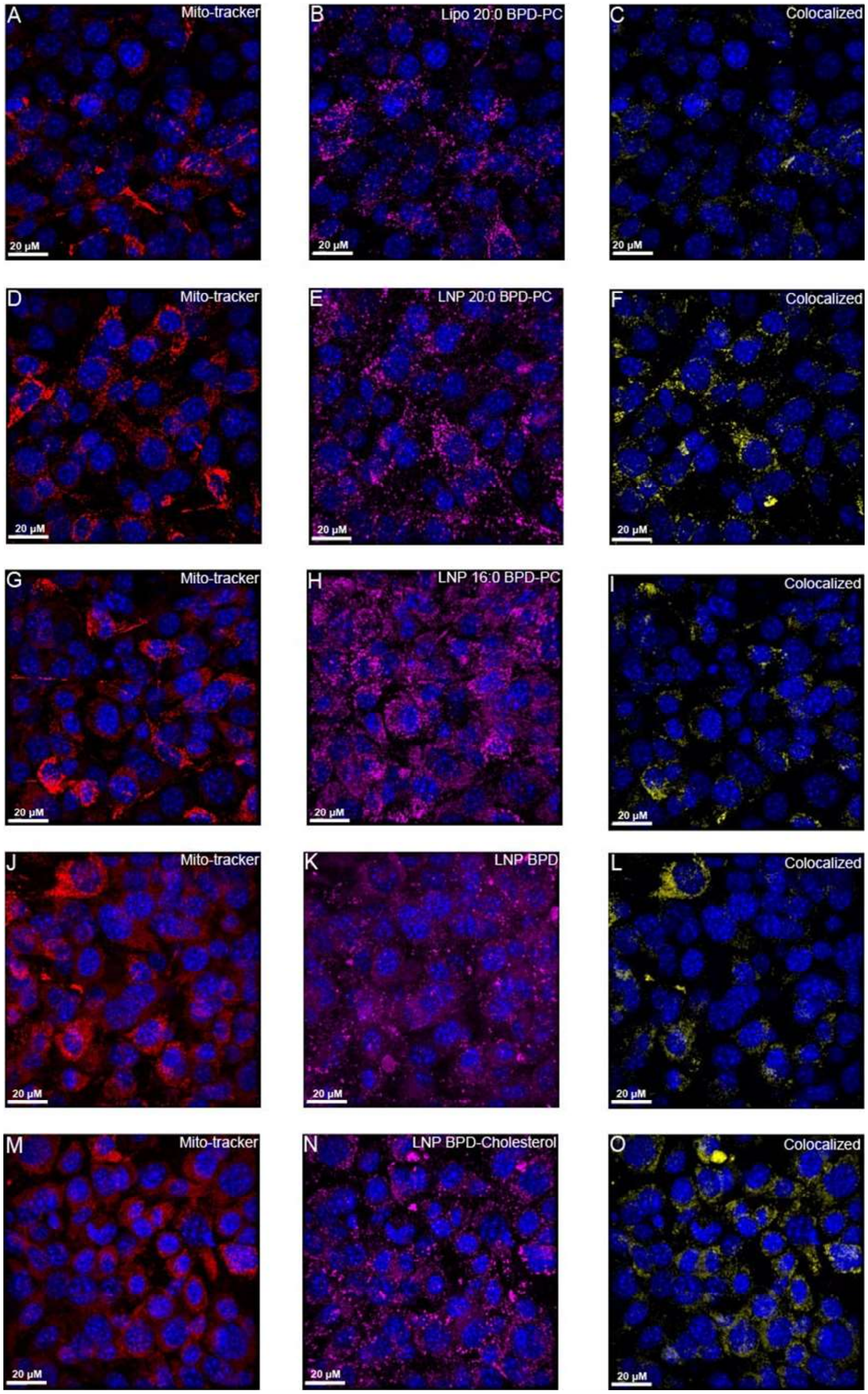

Figure S19: Representative confocal microscopy images of CT1BA5 cells using a 100 × objective after 24 h incubation with Lipo 20:0 BPD-PC (B), LNP 20:0 BPD-PC (E), LNP 16:0 BPD-PC (H), LNPBPD (K) and LNP BPD-Cholesterol (N). Mitochondria are labeled with MitoTracker (red; A,D,G,J,M). For each formulation, the first two images were merged using Imaris software to identify regions of colocalization (yellow; C, F, I, L, O) between the formulations and mitochondria. The brightness of the images was adjusted for improved visualization but all quantitation of co-localization was performed on unmodified images. Scale bars are 20 µm.

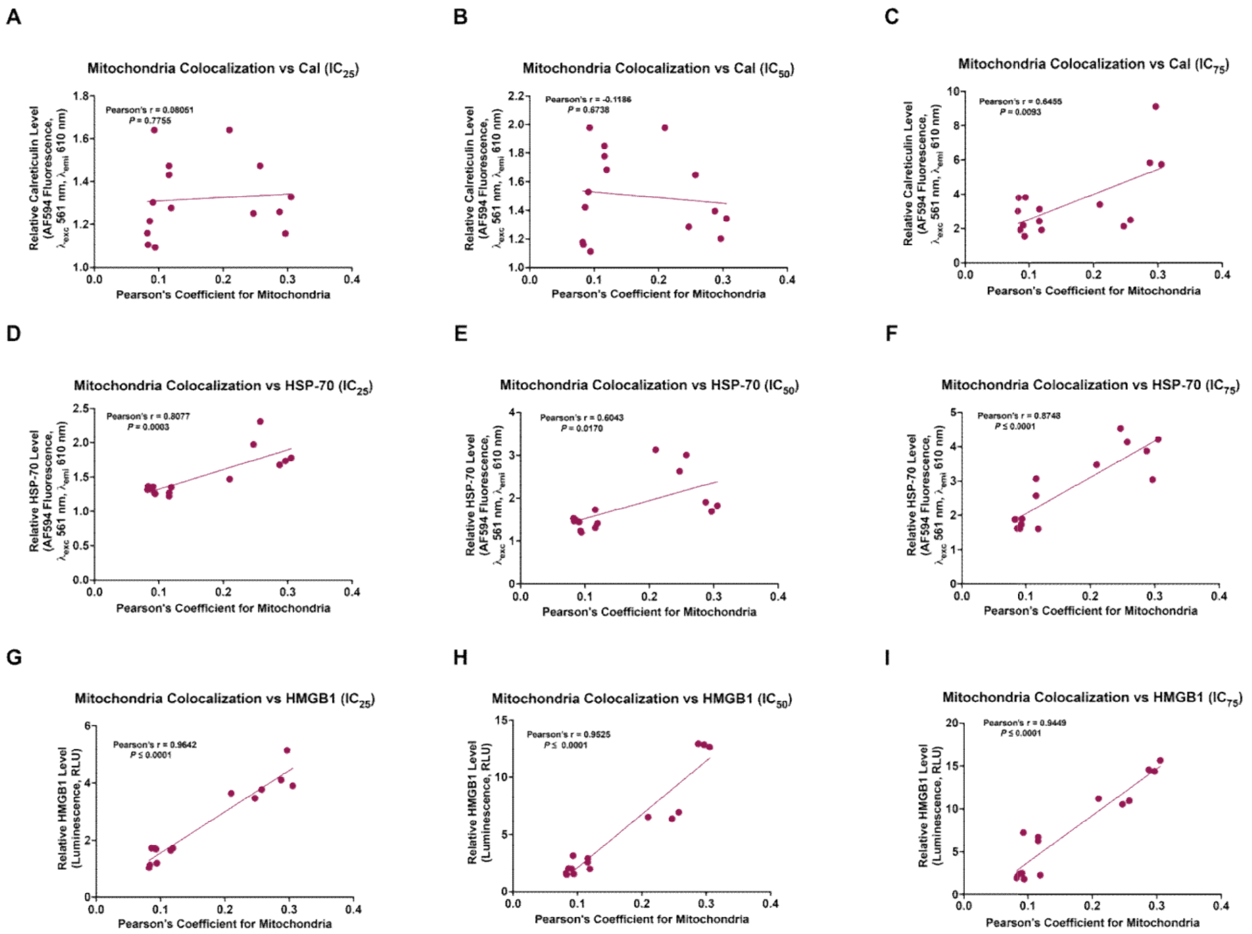

Figure S20: Scatter plots representing the relationships between the exposure of the immunogenic cell death markerscalreticulin (Cal), HSP-70, and HMGB1 in CT1BA5 cells when using IC_{25}, IC_{50} and IC_{75} BPD equivalent concentrations of V-LNPs and the Pearson's coefficient obtained for their co-localization with MitoTracker.

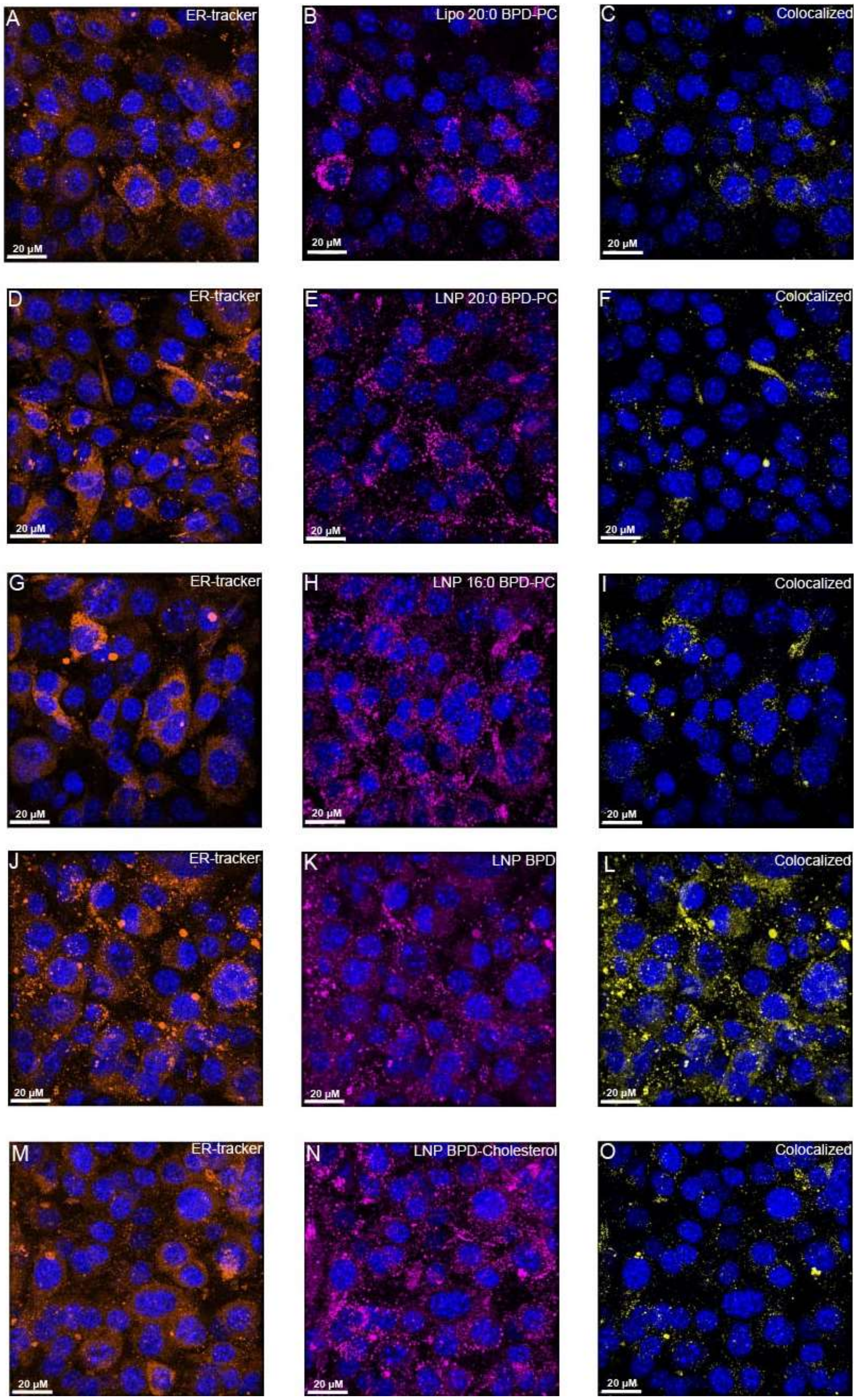

Figure S21: Representative confocal microscopy images of CT1BA5 cells using a 100 × objective after 24 h incubation with Lipo 20:0 BPD-PC (B), LNP 20:0 BPD-PC (E), LNP 16:0 BPD-PC (H), LNPBPD (K) and LNP BPD-Cholesterol (N). Endoplasmic reticulum are labeled with ER Tracker (orange; A,D,G,J,M). For each formulation, the first two images were merged using Imaris software to identify regions of colocalization (yellow; C, F, I, L, O) between the formulations and endoplasmic reticulum. The brightness of the images was adjusted for improved visualization but all quantitation of co-localization was performed on unmodified images. Scale bars are 20 μm.

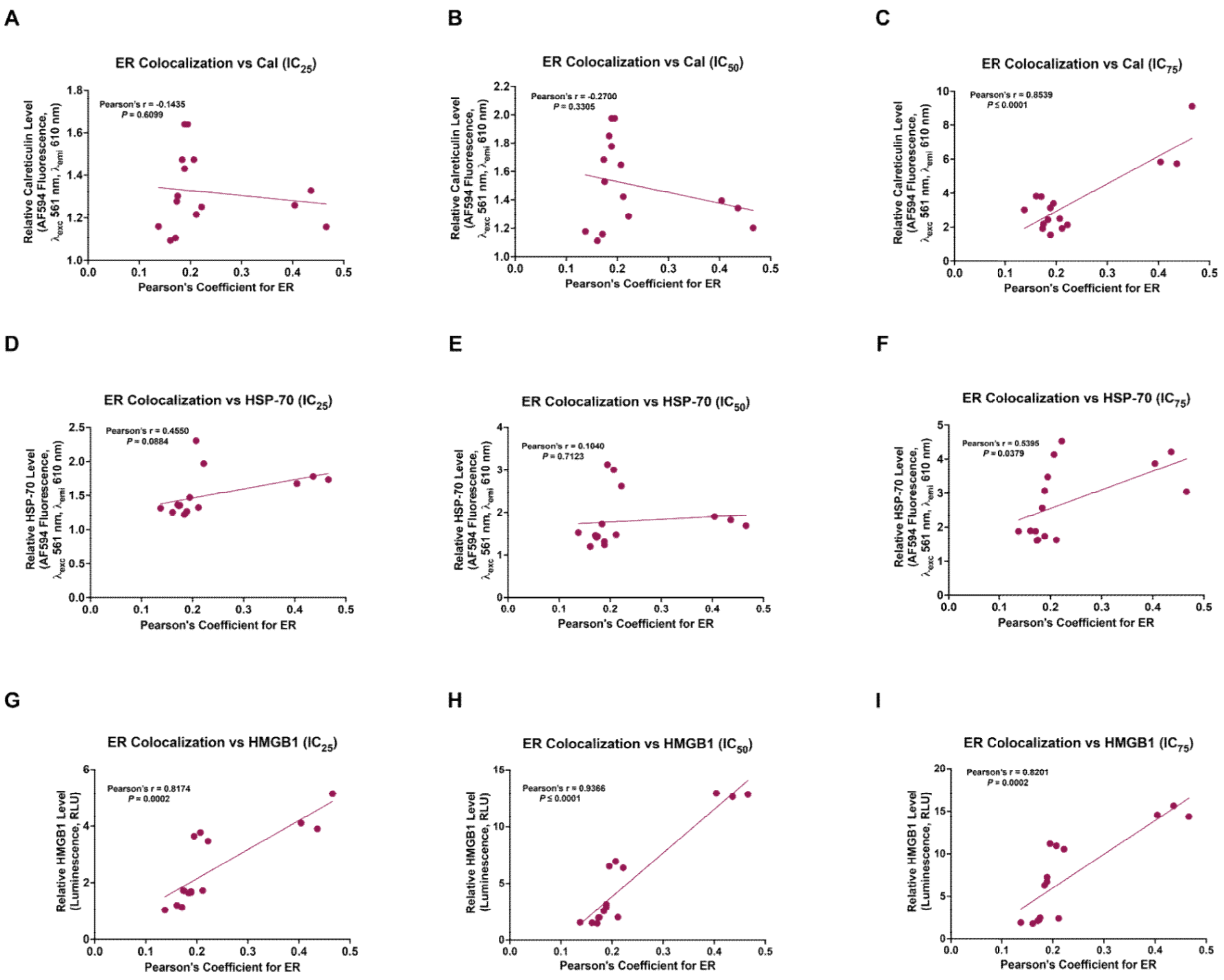

Figure S22: Scatter plots representing the relationships between the exposure of the immunogenic cell death markerscalreticulin (Cal), HSP-70, and HMGB1 in CT1BA5 cells when using IC_{25}, IC_{50} and IC_{75} BPD equivalent concentrations of V-LNPs and the Pearson's coefficient obtained for their co-localization with ER Tracker.

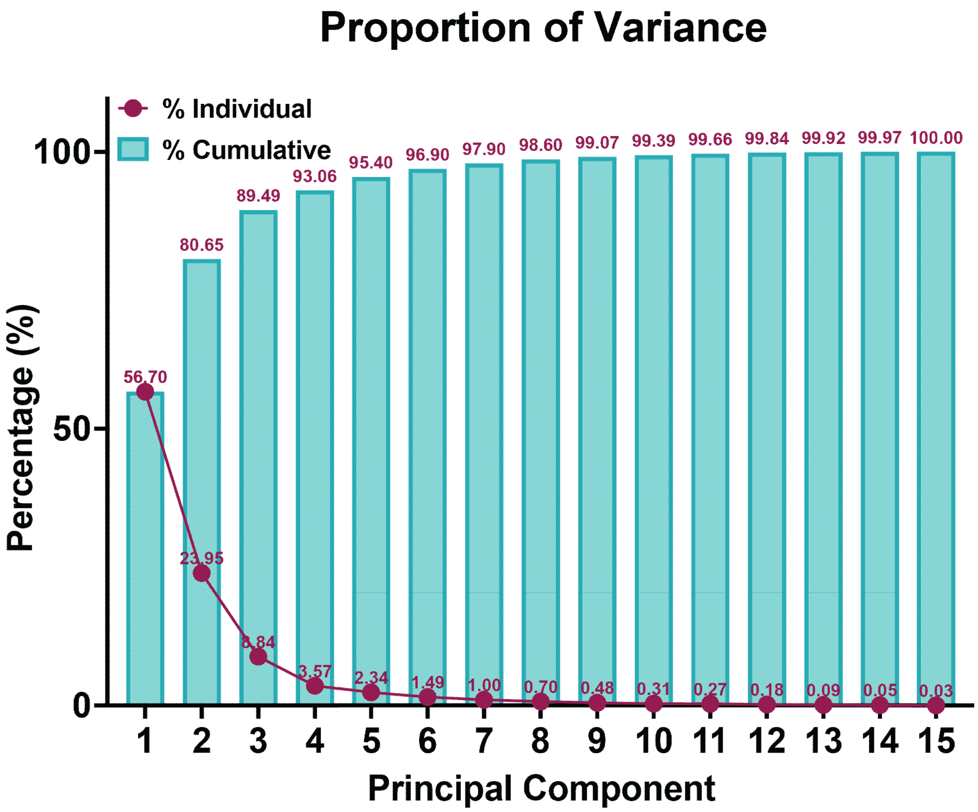

Figure S23: Proportion of variance explained by each principal component (PC). The bar graph represents the individual variance contribution (%) of each PC, while the line graph shows the cumulative variance (%) across the components. PCA analysis was conducted using GraphPad Prism v10.4.1.

References:

[1] G. Obaid, W. Jin, S. Bano, D. Kessel, T. Hasan, *PhotochemPhotobiol* **2019**, *95*, 364–377.

[2] I. Rizvi, G. Obaid, S. Bano, T. Hasan, D. Kessel, *Lasers Surg Med* **2018**, *50*, 499–505.

[3] I. Rizvi, S. Nath, G. Obaid, M. K. Ruhi, K. Moore, S. Bano, D. Kessel, T. Hasan, *PhotochemPhotobiol* **2019**, *95*, 419–429.

[4] M. Guirguis, C. Bhandari, J. Li, M. Eroy, S. Prajapati, R. Margolis, N. Shrivastava, K. Hoyt, T. Hasan, G. Obaid, *Nanophotonics* **2021**, *10*, 3169–3185.

[5] C. Bhandari, A. Moffat, N. Shah, A. Khan, M. Quaye, J. Fakhry, S. Soma, A. Nguyen, M. Eroy, A. Malkoochi, R. Brekken, T. Hasan, J. Ferruzzi, G. Obaid, *Adv Healthc Mater* **2024**, 1–26.

[6] N. Shah, S. R. Soma, M. B. Quaye, D. Mahmoud, S. Ahmed, A. Malkoochi, G. Obaid, *ACS Appl Bio Mater* **2024**, *7*, 4427–4441.

[7] B. Li, M. Zhao, W. Lai, X. Zhang, B. Yang, X. Chen, Q. Ni, *AngewandteChemie - International Edition* **2023**, *62*, DOI 10.1002/anie.202302676.

[8] Y. Mo, M. H. Y. Cheng, A. D'Elia, K. Doran, L. Ding, J. Chen, P. R. Cullis, G. Zheng, *ACS Nano* **2023**, *17*, 4688–4703.

[9] L. Zhang, K. R. More, A. Ojha, C. B. Jackson, B. D. Quinlan, H. Li, W. He, M. Farzan, N. Pardi, H. Choe, *NPJ Vaccines* **2023**, *8*, 1–14.

[10] H. Huang, Z. Wang, Y. Zhang, R. N. Pradhan, D. Ganguly, R. Chandra, G. Murimwa, S. Wright, X. Gu, R. Maddipati, S. Müller, S. J. Turley, R. A. Brekken, *Cancer Cell* **2022**, *40*, 656-673.e7.

[11] J. Li, K. T. Byrne, F. Yan, T. Yamazoe, Z. Chen, L. P. Richman, J. Lin, Y. H. Sun, A. J. Rech, D. Balli, A. Hay, Y. Sela, A. J. Merrell, S. M. Liudahl, N. Gordon, J. Robert, S. Yuan, S. Yu, T. Chao, S. Ye, T. S. K. Eisinger-, R. B. Faryabi, J. W. Tobias, S. Lowe, L. M. Coussens, **2019**, *49*, 178–193.

[12] S. R. Hingorani, L. Wang, A. S. Multani, C. Combs, T. B. Deramaudt, R. H. Hruban, A. K. Rustgi, S. Chang, D. A. Tuveson, *Cancer Cell* **2005**, *7*, 469–483.
